# Spirit of the Foreign Periodicals, &c.

**Published:** 1838-10-01

**Authors:** 


### Spirit of the Foreign Periodicals, &c.

### Some Observations on the Changes produced in the Blood by Medicinal Substances. By C. G. Mitscherlich.

An enquiry into the action of sulphate of copper on the animal system occasioned Dr. Mitscherlich to seek for copper in the blood of animals poisoned with this salt, and also to observe the blood with the microscope. The experiments and observations instituted for the latter purpose required a comparison with the changes produced in the blood by other medicinal substances and poisons, and thus a series of experiments commenced, which, though not yet ended, have yielded some results, which the author now communicates.

In order to ascertain the effects of medicinal substances, and particularly to trace these effects step by step, it is first necessary to know the chemical combinations, which are formed by a medicinal substance in the stomach, &c., and to determine which of these can be absorbed. Then the changes which the blood may undergo in this way, and which it does actually undergo in the economy, are to be ascertained. In order to know these, the habitude of the solution of a medicinal substance in water with respect to blood, the habitude of the same medicinal substance in its combination with the albuminous material, &c., which may be formed in the stomach, and lastly, the habitude of this medicinal substance in living animals, and in man, with respect to the change which the blood undergoes, are all to be investigated. The sulphate of copper, and the sulphate of iron, whose combinations with albumen may be readily exhibited, and in which the composition of the latter combinations is already ascertained by chemical investigation, were chiefly employed for the following experiments. The blood-globules in the frog are peculiarly adapted on account of their size and flat form for this series of experiments, and in the frog one can likewise observe the changes of the blood in poisoning. One of Schiek's microscopes with a 400 magnifying power gave the following results.

The concentrated solution of sulphate of copper in water, mixed with some frogs' blood, first presented a turbidness of the serum, which depends on the formation of insoluble combinations. The blood-globules retain their oval form, but often appear of an irregular figure, being sometimes more, sometimes less curved on the surface. If one observe the blood-globules when they stand on the edges, or turn round, they appear so thin, that one cannot see the muscles, and because they are curved on the surface, they present great irregularity in the direction of the edge. The solution of the sulphate of iron in water is similarly disposed with respect to the blood-globules. This change of form probably depends on the chemical action of the metallic salt on the integuments, but probably the contents of the blood-globules is at the same time diminished and changed. If the above solutions be injected into the veins, a portion of the blood must be changed in the way now mentioned. In other solutions it is observed that the blood-globule swells in volume, which appears to be occasioned by the admission of the surrounding fluid. There is further observed, in concentrated solutions of other substances, a great multiplicity of form in the blood-globules, which have not yet been established by the author, and which possess but little value, as long as the connexion of the new form with the structure of the blood-globules, with the attraction of both fluids, as well as with the chemical relation of the constituents of the solution and of the blood-globule cannot be demonstrated. Some changes of form indicate that one may be able to infer from them the structure of the blood-globules.

The change of form, which the solutions of sulphate of copper, or of sulphate of iron, of sal ammoniac, chloride of sodium, saltpetre, &c. produce, does not take place in the living system, nor does the acetic or oxalic acid, nor ammonia, &c. dissolve the blood-globules, if these medicinal substances are not immediately injected into the blood-vessels. In the stomach, to wit, or in other organs, new chemical combinations are formed before absorption, and none of these substances passes unchanged into the blood. In order to discover changes of the blood-globules in the way they may take place in the system, the blood must be mixed with solutions of the combinations which may be formed in the stomach, and from hence be again absorbed, Our author first experimented with the sulphate of copper and albuminous material, dissolved this combination in the smallest quantity of hydrochloric acid, and mixed the compound with a small quantity of blood. When observed on the surface, the blood-globules were for the most part rounder and somewhat smaller; placed on the edge, they appeared considerably increased in thickness, so that the thickness amounted to from ¼ to ½ in length. The nucleus was likewise increased, and, if the blood-globule stand on the edge, the nucleus is seen in the centre, without its touching the lateral parietes, so that the external covering every where surrounds the nucleus without being touched by it, and according to the quantity of the fluid which it incloses, it lies nearer to or farther from it. The contents of the blood-globules might probably amount to from twice to four times the quantity which they commonly contained in the serum of the blood. The solution of the sulphate of the albumino-oxide of iron in the smallest quantity of hydrochloric acid evinced precisely similar habitudes. This combination consists, according to a chemical examination, of 65 per cent. of neutral sulphate of iron and albumen, and accordingly its position is precisely similar to that of the above combination of the copper-salt.

These experiments only shew that a change of form in the blood-globules is possible under certain circumstances, and that the contents of the same may increase or decrease, but they do not shew that such a change takes place in the living system. With respect to the latter point our author instituted some experiments. Some frogs were put into glass vessels, which contained so much of a weak solution of chloride of sodium, saltpetre, or salamoniac, that the animals were not entirely covered by it. They died within an hour or later, according to the quantity of the salt dissolved. Beneath the skin in the cellular tissue, a great quantity of fluid was found, the blood was dark, and was very slow in coagulating, and the serum existed in very great quantity. A change of form in the blood-globules he was not able distinctly to perceive. If, on the contrary, frogs were placed in a solution of the sulphate of the albumino-oxide of iron, the animals die in about from 24 to 48 hours. Then scarcely any fluid is found beneath the epidermis, the blood is observed to be very thick, clear red, and rapidly coagulating, and the quantity of serum is very much diminished. Neither could an essential change in the form of the blood-globules be detected here. If the blood be examined in frogs after their being exposed for a long time to the action of the above fluid, but before they have died in it, it is found changed in the same sort of way, but not however in the same degree. A change of form in the blood-globules was accordingly not to be found in the above experiments, notwithstanding the serum was essentially changed; on the contrary, the different quality of the serum in the above experiments, is an important point for ascertaining the mode of action of many medicinal substances.

These experiments our author is determined further to extend, and make known the results of them, so far as they yield certain and determinate facts.—(*Archiv. für Anatomie, Physiologie und Wissenschaftliche Medicin. Von Dr. J. Müller, Jahrgang*, 1838. *Heft* 1.)

---

M. RASPAIL ON THOSE DISEASES WHICH MAY BE THE WORK OF INSECTS, AND ON THEIR TREATMENT.

M. Raspail very justly remarks, that medical men have not yet paid due attention to the very curious, but certainly very obscure, subject, how far the presence of parasitical insects in the living body may be the cause of disease, not only in man, but likewise in the lower animals.

So little do we know of the agency of atmospheric phenomena in the production of numerous wide-spread maladies, that it may perhaps be very safely asserted, that in the present day we cannot boast of any information at all more exact and trustworthy than was possessed by the writers of antiquity.

Our attention has been exclusively directed to the study of the merely physical and sensible changes of the air, viz.: those which respect heat and cold, moisture and dryness, gravity, electrical condition, and so forth; and we seem to have scarcely even suspected that there may be other kinds of change and depravation, depending upon the diffusion of innumerable microscopic animalculæ through the atmosphere, although many phenomena in the history of some diseases render such a supposition far from being improbable.

There exists, it is well known, says M. Raspail, an entire class of microscopic animals, known to naturalists by the name of Infusoria, and which were first comprehensively described by the German author Müller. Their name indicates that it is in infusions, or liquids which contain organic fermentible substances, that these living beings are developed. We are not to suppose that the fermentation is owing to the presence of these animalculæ, or that this process is, properly speaking, the *cause* of their development. It is more reasonable to infer that the changes, which the fluid undergoes, are only favourable and necessary to the evolution and metamorphosis of *ovula* or *germs*, pre-existent in, and diffused through, it.

If a small portion of meat is permitted to soak for a few days in pure water, the fluid, when examined with the microscope, will be found to contain innumerable infusory animalculæ. The same is the case if we use a portion of the white of egg, or the leaf of a plant, in the place of the meat. If the albumen be within a vessel or any other tissue of the body, the result will be the same, provided it be in the same conditions; or, in other words, provided it be at rest, exposed to the air, and removed from the *normal elaboration* of the organ. If therefore there be formed in a living tissue an albuminous deposit of pus—which is not fœtid and not yet ammoniacal—there may speedily be developed in it multitudes of infusory animalculæ, of different sorts, according to the nature of the purulent fluid.

The medical journals in the year 1836 announced the discovery of an infusory animalcule in the pus of syphilitic sores, which was regarded by many as the real and essential cause of the venereal disease.

But from the descriptions, which were given of this animalcule, we (M. Raspail) at once recognised that it was altogether similar to, and indeed identical with, the infusoria which are developed in an infusion of meat—in short that it was the *Cercaria gyrinus*, or *gibba* of Müller. We did not hesitate to assert that this animalcule was the effect and not the cause of the disease; that it was the *parasite* and not the *artisan* of the pustules. Perhaps we require no other proof of this statement than the circumstance of no infusoria of any sort being ever discoverable in the pustules when first formed.

M. Raspail remarks that no one can imagine, before he has studied this curious subject with attention, under how many forms, and with what constancy and regularity, the presence of an insect in any tissue of a living body, vegetable or animal, causes certain lesions or organic changes.

It is indeed very wonderful to notice how many new functions—morbid as a matter of course—these minute animalcules cause to arise, either on the surface or in the interior of living bodies. One, for example, residing in the centre of a cellule or little cavity, fashions it into a sphere, as a potter does a vessel of clay; and this atom of a being produces a large and wide-spread *gale* on the leaves of the oak; while another insect, equally microscopic, engenders on the same leaves a sort of little flat eminence, mamillated in the centre, and which has been often mistaken for a species of fungus or mushroom.

A third, by pricking the stem of the rose, causes a minute greenish and mossy-looking production to sprout out, which gradually extends itself, and is known by the name of *bedeguar*. But it is unnecessary to pursue this curious subject, which, although well fitted to throw some light on the development of morbid structures, belongs rather to the botanist than to the physician.

M. Raspail is strongly impressed with the idea that many, if not all, of the Exanthemata are in truth the result of insect operation on the skin; just in the same manner as the diseases of the surface of plants are certainly, in most cases, attributable to this agency. He alludes to the cutaneous affections so frequently caused by gnats, lice, fleas, bugs, and by the *acarus* of scabies, or the itch. If, as is well known, these insects can produce papulæ, pustules, &c. on the skin, is it very unreasonable to suppose that many papular and pustular exanthemata may be the work of minute microscopic animalcules, which have hitherto eluded our research?

Those, who have resided in tropical climates, have probably seen the effects of the *chigres* on the feet of negroes. If these insects are extracted, all the unpleasant effects speedily subside; but if they are permitted to remain in the part, the foot and leg have been known to take on all the characters of aggravated elephantiasis. From this instance, M. Raspail, with rather a hurried zeal, concludes that we should refer the various cases of elephantiasis to the different phases of the invasion of an insect: (donc il faut rapporter les divers cas *d'elephantiasis* aux diverses phases de l'invasion d'une insecte).

Besides exanthematous diseases, M. Raspail is of opinion that several others, such as cancer, tubercles,* cholera, influenza, variola, and certain disorganisations of the liver, kidneys, &c. are the work of entozoa, or insects in the animal economy.

It may be objected to such an idea, in the case of small-pox at least, that the virus of this disease is known to retain its morbific properties, after it has been kept in a dried state for a considerable length of time. But this objection is at once answered, when we call to mind that the ova of numerous insects, after being completely desiccated for months and even years, will speedily become vivified and germinate, if moistened with a little water. Perhaps the circumstance of such a disease as variola affecting the human body only once—at least generally speaking—in the course of a life-time is less easily explicable on the *insect ætiology*, which we have now proposed. But then it is worthy of notice that it is not at all improbable that it is only under certain conditions of the system—the nature of which however, we acknowledge, is not at all known to us—that the presumed animalcules can have any influence.

In conclusion, M. Raspail directs the attention of his readers to the excellent effects of camphor, not only as a preservative against the attacks of all the common vermin, such as lice, bugs, fleas, &c. but also as a most effectual remedy against the itch, and one or two other diseases, which are perhaps the work of insects on the skin. He strongly recommends that a certain portion of this substance should be always mixed with the sulphur ointment used against scabies, and with the white precipitate ointment used against *crabs*, lice in the head, &c.—*L'Experience.*

---

* Several pathologists, at different times, have suggested that tubercles are at first mere *hydatids* or *acephalocysts*. M. Raspail thinks that it is more probable that these morbid products are the work or effects of animalcules, than that they are the animalcules themselves.

### Scientific Notices in Natural History.*

### 1. *A new Classification of the Entozoa found in the Human Body.*

| | | | |
|---|---|---|---|
| Infusoria. | 1. | Cercaria Seminis | in the semen. |
| | 2. | Trichina Spiralis | —— voluntary muscles. |
| Sterelmintha (parenchymatous) | 3. | Echinococcus hominis | —— liver. |
| | 4. | Cysticercus cellulosæ | —— muscles, brain. |
| | 5. | Ditto visceralis | —— intestines. |
| | 6. | Tænia solium | —— small intestines. |
| | 7. | Bothriocephalus latus | —— ditto, ditto. |
| | 8. | Polistoma venarum | —— veins. |
| | 9. | Ditto pinguicola | —— ovaries. |
| | 10. | Distoma hepaticum | —— gall-bladder. |
| Nemetoidea (having vessels & nerves.) | 11. | Ascaris vermicularis | —— rectum. |
| | 12. | Ditto lumbricoides | —— small intestines. |
| | 13. | Strongylus gigas | —— kidneys. |
| | 14. | Spiroptera hominis | —— bladder. |
| | 15. | Tricocephalus dispar | —— cæcum and large intestines. |
| | 16. | Filaria bronchialis | —— bronchial glands. |
| | 17. | Ditto medinensis | —— cellular texture. |
| | 18. | Ditto oculi | —— eye. |

### 2. *On the Heat or Temperature of Insects.*

It is well known that the interior of bee-hives maintains, throughout the Winter, an elevated temperature. This is no doubt owing to the number of bees collected within a small space. Mr. Newport has recently ascertained, from the results of numerous experiments, that *winged* insects, and more especially such as fly about a great deal, develop much more caloric than those which are *apterous* or *wingless,* as ants, &c. All larvæ and chrysalides also, having a much less active vitality and a less extended respiratory apparatus than perfect insects, have a lower temperature than the latter.

Indeed it seems to be a general law that the amount of animal heat is without exception commensurate with, and proportionate to, the development of the respiratory and digestive functions. Butterflies, when flying about, appear to disengage a greater quantity of caloric, than when they are at rest; and it is known that silk-worms, which eat so voraciously and then require, more than at other times, a frequent renewal of air, maintain a very elevated temperature.

### 3. *Ventriloquism in Birds.*

The Agami of Guiana (*psophia crepitans*), belonging to the stork or crane family of birds, has long been known to have ventriloquist powers; uttering sounds which seem to proceed from the anus, its bill all the while remaining quite closed. Many other birds have a similar faculty; and indeed we might *à priori* have anticipated this from the anatomical peculiarities of the windpipe in this class of animals,—the trachea of birds being provided with a larynx at its lower or bronchial, as well as at its upper, extremity. Hence, by making a continuous inspiration, sounds may proceed from the chest, while the proper or superior laryngeal cartilages

* We do not often depart from our rule of devoting this Journal to subjects which are of strictly practical import. It is interesting however, and useful at the same time, to allude occasionally to some topics in natural history, as they cannot fail to enlarge our knowledge of the physiology of life.

remain at rest. All aquatic birds, are more or less decidedly ventriloquist; so also is the thrush, the black-bird, the red-breast and many others of the warblers.

4. *On the Origin of the Parasitical Cryptogamous Plants of Vegetables and Animals.*

M. Unger has recently endeavoured to shew that the stagnation of the juices in the intercellular spaces of plants is in itself sufficient to produce the pulverulent parasites, known under the names of *uredo, puccinium, æcidium,* &c.

The granula or pulverulent corpuscles, of which these consist, exhibit numerous *sporidia,* simple or multilocular, which fructify and propagate themselves upon the organism on which they are developed. These formations are therefore properly *entophyta* or vegetable parasites, and are strictly analogous to *entozoa* or animal parasites. Some of these are limited to the surfaces only of living beings, and are designated *epiphyta* in vegetables, and *epizoa* in animals.

If these cryptogamous vegetables—and we may add the animalcules, whose origin and growth is equally mysterious —were spontaneous productions, we might reasonably suppose that they would not have any fixed or determinate forms, but that they should be variable and uncertain in all their characters. This however is not the case. The ovules, germs, or sporules of these living beings are so microscopic, and at the same time so pellucid, that they quite elude all attempts to investigate them minutely. It is not therefore very surprising that they should be widely diffused, as in all probability they are transported hither and thither by currents of wind, &c. although they remain quite unappreciable by the human organs of sense. It is a curious subject of enquiry, what are the circumstances which favour, or which are necessary to, the development of these vegetable germs?

We know that many of them will not grow upon any, save a very few, objects. For example, the germs of the *onygena exigua* seem to continue floating through the atmosphere, until they meet with the hoof of a dead horse, the only substance on which they ever develop themselves; and again the elegant *isaria* is never found, except upon the chrysalides of butterflies. This serves to explain the development of the *muscardine* of silk-worms. The species of mouldiness, described by various authors under the name of *botrytis bassiana,* is developed only in the fatty matter under the skin of the silkworm, when diseased. Other species of *mucedo* or mould have been detected on various dipterous and orthopterous insects; and it has been observed that the granules or corpuscles, of which these cryptogamous productions consist, are frequently thrown or spurted out to a considerable distance, and attach and fix themselves to other living beings.

When we consider these things, does it seem improbable that some epidemic diseases, especially those of an exanthematous character, may probably be the effect of cryptogamic vegetations, rather than mere pseudo-morphoses of the skin? We already know for certain that some disorders—the itch, for example—are the direct results of the presence of minute insects. Now we cannot very well believe that these are developed by, what is termed, spontaneous generation; the germs or ova of the animalcules must have been derived from somewhere; but whence, or in what manner they are derived and propagated, we literally know nothing.

5. *Edible Milky Juices of Vegetables.*

The greater number of milky-juiced plants—such as the Euphorbiæ, Apocyneæ, &c.—are acrid and poisonous. Certain members however of these vegetable families yield a safe and nourishing sap. Thus the cow-tree (*galactodendron brosimum*), which belongs to the family of figs (*urticeæ* of Jussieu), is a striking example of this fact. There is a still more wonderful plant of this kind in Guiana, the *tabernæmontana utilis.* It is one of the family of the Apocyneæ, most of the members of which are more or less dangerous. It yields a rich, creamy, and sweet-flavoured juice, which is said to be very nourishing.

M. Poiteau has recently discovered in another of the Apocyneæ, the *Couma Guianensis*, similar properties.

One of the most remarkable instances of a milk-bearing plant is found in the poisonous family of the Euphorbiaceæ. The *Euphorbia Balsamifera* of the Canary Islands is, according to Von Buebe, at certain seasons, so full of a milky juice that its bark or rind becomes quite distended and shining. When a cut is made into it, the milk is thrown out to a considerable distance. The inhabitants collect it and thicken it over the fire into a gelatinous or pasty consistence; and this food they call *tabayba*.

Another species of this family, the *Euphorbia Cananensis*, which is usually found growing in the same localities as the preceding, is well known to yield a most virulent poison. — *Journal de Pharmacie, &c.*

---

### On the Influence of the Cerebellum and Spinal Marrow on Erections of the Penis.

MM. Gall and Serres were among the first to maintain that there exists an intimate connexion between the functions of the cerebellum and the feelings of sexual desire.

M. Bourdon, in his Medical Physiology, published in 1828, has adopted their views. At page 116 he uses these words: "The increase of the cerebellum always announces a greater degree of corporeal vigour, and a greater propensity to sexual intercourse. Moreover, the diseases of this organ, and more especially such as are of an apoplectic nature, are almost uniformly co-existent with affections of the generative parts. MM. Gall and Serres have frequently found effusions in the cerebellum in persons, who had exhibited the phenomena of obstinate priapism or nymphomania."

The truth of this doctrine has however been disputed by numerous able writers, and medical men of the present day seem to be quite uncertain what value should be attached to it.

Perhaps the following remarks will serve to clear the subject of part of its obscurity.

It would seem from the results of pathological enquiry, that all the regions of the cerebellum have not an equal degree of influence on the genital organs. The *processus vermiformis* appears to be the part most intimately and immediately influential. Out of *nine* cases of hæmorrhage of the median lobe of the cerebellum there were six, in which seminal pollutions were observed; whereas in not one of the cases of hæmorrhage occurring in the lateral lobe was this symptom manifested. It would seem that the more near that the effusion, be it sanguineous or purulent, is to the *rachidian bulb*, and the greater that the compression upon this part is, the more decided and permanent is the affection of the genital organs. Of *thirty-six* cases of tumors developed in the substance of the cerebellum, permanent erection of the penis was observed in *one* only; and in this solitary instance, the tumor exercised a very manifest compression on the part alluded to. It is important to bear in mind, that it would seem that the affection of the genital organs is rarely, if ever, present in those diseases of the cerebellum, in which there is any atrophy or decided ramollissement of its texture.

We shall now state a few data, illustrative of the

*Influence of the Spinal Marrow* on the functions of the generative organs.

M. Ollivier is of opinion that, in acute Myelitis, or inflammation of the spinal cord, there is generally a tendency to priapism, and that the chronic form of this disease induces ordinarily an opposite state, or one of impotence. It is a well-known fact that the penis is frequently found to be in a state of erection, even for some time after death, in criminals who have been executed by hanging.

M. Segalas, in 1825, announced to the Royal Academy of Medicine that, according to his experiments, the *vesiculæ*

*seminales* seem to be under the in-influence of the spinal marrow, and that the state of these organs may serve to throw some light on the diagnosis of wounds of this nervous cord. (Archiv. de Med. t. ix.)

The mode of action of those remedies, which have a special operation on the spinal medulla, such as the nux vomica, &c., seems to lead to a similar conclusion. Certain it is that strychnine has succeeded in curing several very obstinate cases of sexual impotence. (*Vide Journal de Connoiss. Med. Chir. May,* 1836).

It is moreover worthy of notice that *douches* of cold water on the lumbo-sacral region have been found very useful in checking involuntary pollution. The same remedy is used with advantage against the nocturnal incontinence of urine in young persons.

The occasional application of leeches, or even of the cupping-glasses, over the same part is beneficial.

From what we have now stated, it appears that the state of the spinal marrow has quite as much, if not a more decided and direct, influence on the generative organs as the cerebellum itself.

Physiology confirms the justness of this remark. Most persons are probably aware that lying on the back is apt to induce priapism. Now the cause of this is doubtless that the heat of the bed excites and stimulates the spinal cord, which then re-acts on the generative organs. Friction on the loins has the same effect in a more powerful degree. Slight irritation or puncture of the medulla spinalis—when this has been denuded in experiments—is reported by M. Segalas to induce fulness and erections of the penis.

These circumstances are perhaps sufficient to prove the direct and immediate influence of the spinal cord on the genital functions. If we now enquire whether the whole extent of this cord appears to be equally influential in this respect, the following memoranda may be read with interest. The conclusion, which is deducible from M. Ollivier's great work, is certainly that involuntary erections are apt to occur in the lesions, whether accidental or traumatic, of this organ at all the different regions of the column, cervical, dorsal, and lumbar;* and here it is worthy of notice that the phenomena seemed to be as frequent in ramollissement and other chronic diseases, as in acute inflammation and after recent injuries, of the part—a point of distinction between the symptoms of disease of the cerebellum, and of that of the spinal marrow.

We are now perhaps better prepared to examine the dependence of the generative functions upon the conditions of the great nervous centres, and to ascertain whether any one part either of the encephalon or of the spinal marrow is specially connected with them.

The act of erection has two physiological sources, according as it is, or is not, associated with sensual desire.

All ideas or mental conceptions must be the acts of the encephalon; and every sensation or perceived impression likewise belongs and is referrible to the brain.

It is quite necessary to distinguish the inclination or desire,—whose seat according to Gall is in the cerebellum—from the merely physical act of erection, which may take place independently of any voluptuous thoughts and without the participation of the will, and which is in fact an *accident* of the venous circulation of the generative organs. This distinction will perhaps serve to explain the difference

---

* It would seem however that M. Ollivier himself does not draw the same inference from his observations as our author is inclined to do, as he (M. O.) expressly says, *la lesion de la maelle ne rend pas raison de l'erection de penis.* It should also be stated that the symptom of priapism is unquestionably of more frequent occurrence in lesions of the cervical than of other parts of the spinal column. It was observed in *five* out of *eleven* such cases reported by M. Ollivier; whereas it occurred in *three* only out of *thirteen,* in which the dorsal portion was the seat of the disease.

in the phenomena of cerebellar apoplexies. In some cases there remains a slight or partial degree of perception and sensibility, and the encephalon continues to react, although more feebly, as in a state of health. Now either the disease exercises—whether by continuity or contiguity—a morbid influence on the vitality of the spinal marrow, or this cord is affected with a simultaneous lesion of function: under either condition, there may possibly be erection.

In more severe cases all sensibility is destroyed, and the spinal marrow is not affected or in any way influenced; and then the apoplexy is not accompanied with the phenomenon in question.

What occurs in dreams justifies the division or distinction, which we have pointed out between the two physiological acts or impressions. Were it otherwise, should not all nocturnal pollutions be accompanied with dreams? But this, we know, is not the case; and such pollutions are very often attributable to an irritability of the spinal marrow, being quite unconnected with any sensual ideas.

The conclusions, which M. Petrequin deduces from his researches, are the following:

1. The erection of the penis cannot properly be considered a pathognomonic symptom of the diseases of the cerebellum, although it may be co-existent with them; it has therefore no positive value as such an indication.

2. This phenomenon depends upon an action of the spinal marrow. The truth of this position is exemplified in traumatic lesions of the spine, in death from hanging, in myelitis, hematorachis, and in the effects of dorsal decubitus, friction or titillation of the back, &c. &c.

3. We should distinguish two sorts of erection, that which depends exclusively on the action of the spinal marrow, and that which proceeds *mediately*, or indirectly, from cerebral reaction. The first is purely spinal and corporeal, while the second is cerebro-spinal, and therefore partly mental. The brain cannot act directly on the generative organs, but only through the intravention of the spinal cord.

4. We should not at once conclude, from the occasional existence of erection in cerebellar apoplexy, that the venereal instinct necessarily resides in this part of the nervous system, especially as we are not aware of the state of the spinal marrow in this very disease.

The erection may exist, and no doubt in such cases does exist, without being associated with any sexual appetite or desire. That the seat of this desire may be in the cerebellum seems however to be not at all improbable.—*Bulletin Medicale Belge.*

---

Researches on the Cause of the Abnormal Auscultatory Sounds in the Large Arteries, &c. By M. Beau.

Various have been the explanations proposed by different writers to account for the phenomena, alluded to above.

M. Laennec, who was the first to direct the attention of medical men to their existence, was of opinion that they were attributable to spasm of the arterial tubes.

This *vital* explanation of a phenomenon, which is simply and purely *physical*, is however manifestly at fault.

Of late years, most physicians have admitted that the sounds in question are the result of a certain vibration, *(fremissement)* of the arterial parietes, induced by the rapid flow of the blood along them; but, as to the manner in which the blood causes this vibration, they are not agreed.

Most however seem inclined to assent to M. Bouillaud's doctrine, that it is owing primarily and essentially to an *exaggerated friction of the arterial parietes by the wave of blood,* as it passes along.

We shall briefly examine this view of the question, before we proceed; and for this purpose we shall allude to the results of several experiments performed by different authors.

1. If we attach the extremity of an arterial tube to the canule of a syringe, and then force a stream of water along it with considerable force, we find that on each stroke of the piston—that is, at each time that the tube is most distended—a distinct vibration is perceptible both by the finger and by the ear; the intensity of the vibratory movement being proportional to the force of the piston's stroke, and to the distention of the arterial canal.

2. If a less quantity of fluid be propelled by each stroke of the piston, so that the tube is less distended, no vibratory motion, as described in the former experiment, can be detected; and the only perceptible phenomenon is a pulsatile heaving, as each wave of water is forced along.

3. Without increasing the force (*etendue*) of the strokes of the piston, it is still possible to reproduce the vibratory movements of the artery. All that is necessary for this purpose is to exercise a certain degree of pressure on the vessel; the vibration will then be perceptible at the contracted point.

From the results of these experiments it would therefore seem that the vibratory movement, and the sound which accompanies it, are indeed produced by the *exaggerated friction of the fluid*, passing along, *against the parietes of the vessel;* and it is not less clear that the cause of this exaggerated friction is the great quantity of fluid, passing along the tube, in proportion to its calibre,—whether the defect of proportion is owing to an augmentation of the wave of the current, or to a contraction of the tube at one point of its *trajet*.

It thus appears that the real cause of the phenomena, to which we have been alluding, is a disproportion between the bore of the vessel and the volume of the current which passes through it.

Let us now briefly examine this subject *pathologically*, and try to ascertain whether these phenomena, when they occur in a state of disease, can be explained on the same principle.

The abnormal *bruits* of arteries may be conveniently arranged into such as are local, and such as are general or diffused.

The former are limited to one point, and arise from a morbid action in the part itself; the latter are, on the other hand, connected with the existence of an affection, which causes them to be perceptible in various arteries at the same time.

The local *bruits* are met with in cases of aneurismal tumor, of varicose aneurism, or whenever there exists any compression of an arterial tube.

The effects of pressure on a large artery in eliciting a blowing or sawing sound may frequently be perceived by simply applying, with some degree of force, the end of the stethoscope over the *trajet* of the vessel.

The same result is produced by the pressure of any contiguous tumor. Thus there was recently in the clinique of M. Fouquier a woman, afflicted with a cancerous swelling of the stomach, the pressure of which on the abdominal aorta caused a distinct *bruit de soufflet* to be heard.

M. Rufs quotes a case of a moveable goitre, where the patient could at will cause a similar bruit in the carotid arteries, by varying the position of his neck.

But perhaps the most remarkable example of this kind is that related by M. Bricheteau in his Clinique de l'Hôpital Necker. It was the case of a woman who died in the wards there, after having exhibited all the chief symptoms of pregnancy, and, among the rest, the *placentary* or *uterine bruit*. On dissection, one of the ovaries was found enormously enlarged: the pressure of the tumor on the iliac artery had given rise to the auscultatory phenomena.*

Let us now apply, in explanation of the general or diffused arterial bruits, the theory on which we have attempted to account for the local bruits—viz., the disproportion between the bore of the blood-vessels and the volume of the current, which is propelled through them.

The diseases, in which these abnormal bruits are most frequently heard at different points of the arterial system, are insufficiency of the aortic valves, plethora, hypochondriasis, chlorosis, &c.

---

* M. Bouillaud has quoted this case in proof of his opinion that the *placentary bruit*, during pregnancy, emanates not from the uterine, but from the iliac, vessels.

1. *Insufficiency of the Aortic Valves.*—The characteristic signs of this morbid state are, *a*, a blowing bruit audible during the second sound of the heart, (it may indeed be prolonged, so as to be heard during the first sound also); it is loudest and most distinct immediately over the seat of the aortic valves. *b*. A blowing bruit in many of the arteries of the body: this is isochronous with the dilatation of the arterial tubes and consequently with the first sound of the heart. It is to be remembered that, along with the state of the aortic valves in question, there is usually co-existent a greater or less degree of dilatation of the left ventricle of the heart. Hence the wave of blood at each systole is greater than in health; and, if the contraction of the ventricle is powerful, there is no obstacle to prevent it (the wave) being propelled entirely along the arteries. Under these circumstances the shock of each impulse against the arterial parietes is sometimes so strong, that it may be actually heard at a little distance from the surface of the body.

2. *Plethora.*—In not a few cases of unusual fulness of the blood-vessels, and more especially if the system is at the time affected with any febrile excitement, a vibratory movement, accompanied with a rushing or blowing bruit, may be perceived along the *trajet* of several of the large blood-vessels.

After what we have said above as to the cause of these phenomena—the disproportion between the volume of the arterial current and the size of the vessels—it is unnecessary to enlarge upon this subject.

3. *Hypochondriasis.*—M. Beau has met with two cases only of this disease, in which the arterial *bruits* were very distinct.

Both patients were adult males; and in both the auscultatory phenomena were strongly marked.

Vertigo and confusion, sparks and flashes of light before the eyes, a feeling as if there was every now and then a detonation within the head, or as if a hammer was constantly at work there, oppression, a sense of strangling suffocation, &c., were the most prominent symptoms. When the attack had passed over, the pulse recovered its ordinary strength and volume, and the arterial *bruits* were no longer perceptible.

It will be observed how the symptoms in these cases closely resembled those of extreme plethora; and even some of the outward phenomena, such as the distended state of the subcutaneous vessels, the reddened eye-balls, the flushed face, &c. would seem to indicate that there may be, under certain circumstances, a temporary expansion, as it were, of the circulating fluid.

4. *Chlorosis.*—Here it may be enquired, *in limine*, how can we attempt to explain the arterial *bruits* in this disease by reference to a superabundance of blood, when it is almost universally admitted that there is a decided deficiency of its mass? But in examining this topic a little more attentively, we may be led to the conclusion that the actual *quantity* of the circulating fluid is not diminished, and that it is rather its *quality* only—being much more watery and less nutrient than in health—which is the pathological character of chlorosis.

There is one feature of many cases to which M. Beau appeals as confirming, in his opinion, the idea that there is no positive diminution in the mass or actual quantity of the blood in the vessels. In every case of the disease, in which he has discovered the arterial bruits, the arteries exhibited a degree of fulness, proportionate to the intensity of the chlorotic symptoms, and to the distinctness of the bruits; this fulness diminishing when the disease begins to be removed, and the bruits cease to be heard. It is quite possible therefore that there may be a plethora arising from an attenuated watery, as well as from a rich and crassid blood; and, if so, we can at once account for the auscultatory symptoms of chlorosis on the principles explained above.*

* It may be worth while observing that Boerhaave, in treating of Chlorosis, has expressly intimated his opinion that there is often no deficiency of the

There is another manner in which we may seek to find a *rationale* of some of the arterial *bruits*. Why may there not be, in certain states of the system, an unusually irritable or contractile state of the blood-vessels themselves, so that, while the mass of the circulating fluid remains unchanged, the calibre of the vessels, through which it passes, is more or less diminished?*

Besides chlorosis, there are several analogous affections, especially such as proceed from large losses of blood, in which the arterial bruits are generally very distinctly perceptible. In all these cases the existence of these bruits coincides with a more than ordinary fulness of the pulse: when this ceases, the bruits become invariably less and less manifest. The pulse indeed is soft at the same time that it is full and quickened, *because* the circulating fluids are serous and superabundant, (la mollesse se joint à la plenitude du pouls, parceque le sang est en meme temps sereux et surabondant). It seems therefore that, in chlorosis and some other morbid states of the system, there is a a *serous polyemia,* which has several characters in common with genuine *plethora* or *sanguineous polyemia.*

Besides the cases already specified, in which the abnormal arterial bruits have been heard by many observers, M. Beau has detected their existence in several instances of *lead colic,* of *engorgement of the spleen,* &c. when the pulse was found at the same time to be very full and rapid. He suspects that they will be discovered in many cases of scurvy also; but in his own practice he has had no opportunities of testing the accuracy of this opinion.

It will naturally be supposed that these abnormal bruits, in the cases now alluded to, although analogous in their general character to those which we have in the earlier part of this paper denominated *bruits de l'insuffisance,* and which are usually present in actively plethoric states of the system, have several points of difference: in truth, they are always less rough or rasping, and are much less strongly vibratory, under the former than under the latter circumstances. In both cases the sounds are more distinct in the arteries near to, than in those more removed from, the centre of circulation. Hence they are always louder over the carotids than over the crurals, &c.—*Archives Generales de Medecine.*

red part of the blood, but only a disproportionately large quantity of its serous portion, in this disease.

* Is not this the very doctrine of Laennec, which M. Beau has already impugned as incorrect?

---

### ON SUDDEN DEATH, OCCASIONED BY THE SPONTANEOUS DEVELOPMENT OF A GASEOUS FLUID IN THE BLOOD, AND ITS ACCUMULATION IN THE HEART.

In our last number (see the second last article in the Foreign Periscope) we considered some of the most common causes of sudden death, which have their seat in the encephalon, the lungs, and in the heart and great blood-vessels.

We then promised to continue the subject, and to explain to our readers some recent opinions of M. Ollivier respecting the spontaneous extrication of a gaseous fluid from the blood itself as a cause, hitherto unsuspected, of sudden death.

He premises his remarks by alluding to the total absence of any lesion or pathological chance, discoverable on dissection, in not a few cases of this description; and from this fact he very properly argues that "it is not always in an appreciable organic lesion that the determining cause of death is to be found."

The pathological phenomenon of the presence of an aerial fluid in the cavities of the heart and great blood-vessels—capable as this state is of inducing almost instantaneous dissolution—has very probably escaped the notice of most anatomists in several cases, where no satisfactorily decisive traces of disease have been discovered.

It appears however to have been noticed by some. Thus, Morgagni has narrated a case of sudden death which he attributes to the interruption of the circulation by the presence of a gaseous fluid in the blood; and there seems to be some allusion to such an occurrence even in the writings of Hippocrates.—(*De Sed. et Causis Morbor. Epist* 18, 19, 24.)

The following two cases were recorded, some years ago, in the Dictionnaire de Medecine, art. *Air,* by M. Ollivier.

*Case* 1. A young child, who had been affected with measles for a few days, seemed to be quickly recovering from the disease, when, all of a sudden and without any premonitory symptom, he fainted, and expired in the course of a minute or two. On dissection, the heart and large blood-vessels were found distended with air, the parietes of the heart were emphysematous, and its cavities quite empty of blood. In the course of several hours after death, the emphysema had diffused itself through the subcutaneous cellular texture of the trunk.

No organic change could be discovered in any of the viscera; and there was no trace of putrefaction.

*Case* 2. Similar phenomena to those recorded in the preceding case were observed by M. Ollivier in the body of a robust man, who died quite suddenly a few minutes after he had retired to bed in seemingly perfect health.

The general emphysema did not appear till about twelve hours after dissolution; but then there was not the slightest sign of the putrifying process having commenced.

*Case* 3. A young woman, 22 years of age, was recovering from a fever, which had left her for a length of time very weak. One afternoon, after having been engaged during the day with trying on some masquerade dresses, she had laid herself down on the bed to refresh herself, expecting to be fatigued with the entertainment in the evening.

While attempting to rise to put on her clothes, she suddenly felt extremely faint; her head fell forwards on her chest; she screamed out "*I am dying,*" and expired almost immediately in the arms of an attendant.

M. Ollivier was ordered by the public authorities to examine the body. There was no outward appearance of any injury or violence; the expression of the features was calm, and like that of one asleep.

The encephalon appeared to be quite healthy; the blood, however, which flowed from the divided vessels was frothy from the admixture of bubbles of air.

All the abdominal and pelvic viscera were sound. So also were the lungs.

On opening the pericardium, the right cavities of the heart were observed to be remarkably distended, and, as it were, blown out, so that when tapped with the handle of the scalpel, they sounded hollow, like the stomach.—When an incision was made through its parietes, they at once sunk down: a large quantity of bloody froth was found in the right cavities. The pulmonary artery also contained some frothy blood.

The very peculiar circumstances of this remarkable case of sudden death—their analogy with the phenomena which have been observed in persons who have suddenly died from the accidental introduction of air into a divided vein, and the presence, discoverable on dissection, of a quantity of gaseous fluid within the right cavities of the heart—led M. Ollivier to attribute it to the spontaneous development and extrication of air from the blood itself, while still circulating in the vessels; and such was the professional opinion which he gave in to the judicial authorities.

M. Ollivier, after minutely reporting the particulars of the preceding case, proceeds to state the opinions of various authors on the occasional presence of extricated air in the heart and blood-vessels during life.

Mery* was of opinion that the atmospheric air could pass from the minute cells of the lungs into the pulmo-

* Mem. de l'Acad. des Sciences, 1707.

nary veins. M. Littre* thought that whenever air was discovered in the heart or blood-vessels on dissection, it was almost invariably a *post-mortem* phenomenon, attributable to the arrest and stagnation of the fluids, which had been during life kept in constant circulation. He and also Morgagni admitted, however, that in a few rare cases air might possibly enter into the blood in the manner indicated by M. Mery.

Bichat† seems to have entertained no doubt as to the reality of the occasional development of an aerial fluid in the blood. His words are—"The admission of air into the blood-vessels takes place sometimes in the human subject without being accompanied by the infiltration of it into the cellular tissue: in such cases the death is always very sudden." He adds that he had examined the body of a person who had died almost instantaneously during a convulsive attack of the pectoral muscles, and in whose arteries and veins, more especially those of the neck and head, a quantity of frothy blood, mixed with bubbles of air, was found.

Hitherto it has not been satisfactorily determined, what is the exact chemical nature of the air found in such cases as we have been alluding to.

M. Rerolle,‡ indeed, has recently directed his attention to this subject of enquiry, and the results of some experiments have led him to believe that the air present in the blood-vessels in some cases of profuse hæmorrhage is atmospheric air, and is derived from pulmonary absorption, and not from a direct admission into the divided vessels. He has not however made any series of experiments on the subject.

The important researches of M. Magnus|| having proved that the carbonic acid exhaled during expiration exists already formed in the venous blood, and is not developed in the lungs, will naturally lead us to suspect that the air occasionally found in the heart and blood-vessels may consist, more or less entirely, of this gas.

The determination however of this point is still *sub judice*.

Whatever be the nature of the gaseous fluid, and from whatever cause its development and extrication in the blood may proceed, we may be quite assured of the fact, that whenever it does take place, death is induced almost as rapidly as in the cases in which the air is admitted into the veins during some surgical operations.

Most authors seem to agree that the immediate cause of death in both sorts of cases is owing to a sudden interruption of the circulation, in consequence of the forcible distention of the parietes of the ventricles of the heart.

Bichat indeed, and more recently M. Leroy also, attributed the fatal influence partly to the noxious influence of the air within the cerebral blood-vessels on the brain; but this explanation is of very questionable truth.—*Archives Generales.*

---

### M. VELPEAU ON THE ACCIDENTAL INTRODUCTION OF AIR INTO THE VEINS.

In the Foreign Periscope of our last number, when treating of the report of M. Bouillaud on M. Amussat's memoir on the above subject, we intimated our intention of giving a condensed statement of M. Velpeau's sentiments, as expressed in a long and very elaborate letter, which he has addressed to the editor of the Gazette Medicale.

He remarks:—"Long before the attention of pathologists had been directed to the occasional introduction of the external air into divided veins, cases of sudden death during certain operations were well known to all experienced surgeons. The accident was attributed sometimes to the loss of blood, at other times to exhaustion from the severe pain, or to syncope, fright, &c. In my

---

* Mem. de l'Acad. des Sciences, 1707.

† Recherches physiol. sur la vie et la mort.

‡ Dissertation sur un nouveau genre de pneumatose qui se developpe à la suite des hæmorrhagies. Paris, 1832.

|| Memoire sur les gaz continus dans le sang, et sur la theorie de la respiration.—*Journal de Chimie, Nov.* 1837.

own practice, I have known patients to expire during the attempts of a surgeon to extirpate a diseased thyroid gland, or a large tumor from the axilla, and also immediately after the performance of tracheotomy, when the death was unhesitatingly referred to one of the causes, now mentioned. But, of late years, much stress has been laid on the introduction of the external air into some of the divided veins, and its consequent admission to the heart, as one of the most frequent and rapidly-fatal causes of such an accident. I have carefully collected the reports of all the cases which have been published as instances of this occurrence in the various journals, domestic and foreign, for the purpose of comparing them together, and of examining the accuracy of the conclusions which have been drawn from them. They amount in number to *forty*."

(A short detail of each is given in M. Velpeau's memoir, and the source from which each report is derived. We cannot afford space to extract the particulars, and shall therefore content ourselves with giving the general conclusions which he has drawn from their examination.)

M. Velpeau does not hesitate to state that several (4) of these cases are quite unsatisfactory, and cannot fairly be adduced as examples of the accident. He therefore dismisses them at once from consideration.

In *fifteen* other cases, the patients recovered more or less quickly from the presumed accident. Some of these cases too are of very questionable accuracy. The external jugular vein only was wounded in four of them; and in other two, the mammary veins only were divided. Perhaps therefore we cannot recognise more than *seven* cases in all of this group, as being probable or possible examples of the accident in question.

In the *third* group—containing such cases as proved fatal, but in which no post-mortem examination took place—we find six cases in all. They are recorded by Messrs. Warren, Clemot, Barlow, Goulard, Klein, and Maugéis.

In these six cases we observe that, in M. Clemot's case, the wounded vein was not accurately determined; in Mr. Barlows's it was the internal jugular; in M. Klein's it was the thyroid plexus; in M. Goulard's it was the axillary, and in Mr. Warren's it was the subscapular. The median vein in the fold of the arm was the only one wounded in M. Maugeis' operation.

The *fourth* group comprises such cases as proved fatal, and in which an examination took place after death. They amount in all to seven; and are recorded by MM. Piedagnel, Dupuytren, Delpech, Castara, Ulrich, Roux, and Pategnat. We must however, exclude the case related by the last-named gentleman, as the details which he gives are quite unsatisfactory, and rest on the authority of another person.

In the cases of MM. Piedagnel and Dupuytren, it would seem that the divided vein, by which the air entered, was the external jugular.

In those, which occurred to MM. Roux and Delpech, it was the axillary vein, the operation in both cases being amputation of the shoulder-joint; in Mr. Warren's and M. Castara's cases, it was the subscapular vein; and in M. Ulrich's case it was the internal jugular.

If we now examine, says M. Velpeau, the particulars of those cases which have occurred in the human subject, and compare them with the results of the experiments which have been made on lower animals, and wherein they have died by the artificial introduction of air into the veins, we are forced to infer either that these experiments are incomplete and delusive, or that the cases, alleged to have occurred in the human subject, are unsatisfactory and inconclusive.

It was found that on all occasions a considerable quantity of air was required to kill a dog; that the introduction of the air happened only when a large opening was made in either the internal jugular, the subclavian, or the axillary vein; and that, when the animal died, the heart was always found distended with a frothy fluid, consisting evidently of blood and bubbles of air.

On the other hand, in several of the cases, related as examples of the accident in the human subject, the divided veins were the mammary, the subscapular, the external jugular, or even the facial. In not a few of the cases, the opening in the vein was very small, and the quantity of air admitted must have been very inconsiderable. And lastly, in no post-mortem examination have the same appearances been detected in the human subject, as in the lower animals, which have been the subjects of the experiments.

I would not however have it to be inferred from these remarks that I am of opinion that death from the introduction of the external air into the veins has never occurred in the human subject. All that I am contending for is, that such an occurrence has never been *incontrovertibly proved or demonstrated.*

In several of the cases alluded to above, it is exceedingly probable that it did happen, and that it was in fact the cause of the death of the patient; and in perhaps three or four (including the one recorded by M. Dupuytren) we should say that it did *really* take place. Still, however, we require further data to set the question at rest. Even in M. Dupuytren's case—which on the whole is perhaps the most conclusive—a very few bubbles only of air were found in the heart, upon dissection. But it is to be remembered that a much less serious lesion may prove fatal to the human subject, than to any of the lower animals. The operation of fear, alarm, and mental excitement must necessarily have a very considerable influence on the one, while the other is almost quite exempt from such agency or influence.

In conclusion, we may state that, while we regard the admission of air into the veins during certain operations as a very probable cause of the sudden death, which sometimes happens; science requires more numerous and accurate data on the subject, before we can with confidence draw any fixed conclusions.—*Gazette Medicale.*

---

QUESTION OF THE LIFE AND VIABILITY OF AN INFANT, CONSIDERED MEDICALLY AND JUDICIALLY. By M. MARC.

On the 28th of April, 1834, a lady, who was presumed to be in the eighth month of pregnancy, was suddenly seized with convulsions, which rapidly proved fatal.

Within a short time *(peu de tems)* after dissolution, the Cæsarian operation was performed, and the child was extracted.

The question, that came to be disputed, was whether this infant was alive and therefore capable of inheriting its mother's property, or whether it was dead, at the moment of extraction.

The exhumation and inspection of the child were not made till a month after the period alluded to.

The tribunal consulted MM. Marjolin, Roux and Marc—three of the ablest practical men in Paris—and by them the following report of their opinion was returned.

It may be well, however, to premise a few short particulars derived from the evidence of the different witnesses examined on the trial.

The surgeon, who performed the operation of extracting the child, stated, that he was called into consultation with Dr. Brunard, the physician in attendance, on the 28th of April, at noon; that he remained with her till about two o'clock, and then left; that the patient was then in extreme danger from violent cerebral convulsions; that he was resummoned about four o'clock, but that he did not reach the house till nearly a quarter of an hour after the lady had died; that he immediately, while the corpse was still warm, proceeded to extract the child by the Cæsarian operation; that he distinctly remarked by the elevation of the parietes of the thorax that it, the child, breathed, although the respiration was certainly very feeble; that he noticed also well marked pulsations in the umbilical cord, both before and after it was divided; and moreover, that he was able to distinguish the beats of the heart, when he laid his hand on the left side of the child's chest. He also deposed that the child, when put into a warm-bath, moved its right arm towards its head, and made one effort at inspiration.—The period, during which he was of opinion that life continued, was about five minutes. There was no external lesion or malformation on the body of the infant, and its size indicated that it was probably eight months old.

The next witness, a woman who was present when the lady died, gave similar testimony to that of the surgeon, confirming it in all the essential particulars. It is right to state, that the infant was immediately baptised on being taken from the uterus, as the attendants were all of opinion that life was present.

The evidence, contradictory of the preceding statements, was as follows.

M. Brunard, a physician, deposed that he was summoned to the assistance of Mad. Gallois, the deceased, at four o'clock in the morning of the day on which she died, and that he remained with her till eleven o'clock of the forenoon. He stated that he had not assisted at the extraction of the child, so that he could not say whether it exhibited any signs of life; but that he had been strongly impressed with the idea during his attendance that the child was dead, before the mother ceased to live. He was of opinion that pregnancy had not advanced beyond the sixth month.

Another witness deposed that he was present in the room, when the infant was put into the warm-bath, but that he could not perceive any signs of vitality in it.

We have already mentioned that the examination of the body of the infant did not take place till 31 days after the date of the delivery. It is unnecessary to dwell upon the particulars of the dissection. Suffice it to say, that one lung sunk immediately in water, and that the other one only partially floated.

The *hydrostatic test* seemed to be therefore unfavorable to the idea that respiration had ever been fairly established.

We come now to state briefly the written opinions of the leading authorities in Paris, who were consulted on this important case. The first was M. Velpeau.

After briefly recapitulating the most prominent facts deposed to by M. Cabaret the surgeon, and alluding particularly to the circumstance of the circulation in the cord being still quite distinct after extraction, he at once concludes that *the child was certainly not dead.* He admitted however that the question as to respiration having ever been established cannot be easily determined. The mere circumstance of the lungs being not uniformly buoyant, should not, he thinks, influence us at once in giving an answer in the negative; *as an incomplete inspiration*—especially in an infant prematurely born—*does not always introduce, or does not always leave, a sufficient quantity of air into the pulmonary canals and cells, to render these organs natant.*

He was therefore of opinion that the child was born *viable,* (capable of life,) and alive; and the only point on which he was at all doubtful, was as to the act of respiration having ever taken place.

The next authority is M. Orfila, the distinguished professor of legal medicine in Paris. He arrived at very different conclusions from those laid down by M. Velpeau. He was satisfied, he said, that true respiration had never been fairly established; and his opinion on this point was confirmed by the very circumstance of the circulation through the umbilical cord having been so distinct, as M. Cabaret the surgeon stated it to have been; for, continues he, we well know that whenever a new-born infant begins to breathe freely, the pulsations in the cord speedily become more and more indistinct. Everything therefore, he adds, leads me to believe that respiration was never truly established, and that the pulsations of the cord are to be regarded as depending on the remains of intra-uterine life.

Baron Dubois agreed with M. Orfila in the view which he took of this case. His *consultation* is so brief and precise, that we shall give it in full.

"I have carefully perused all the documents on this important question, and I have particularly examined with the greatest attention the evidence of the professional witnesses. I do not consider that it is at all necessary for me to recapitulate the conflicting opinions, and the contradictory statements in the different papers submitted to my inspection. It is sufficient to affirm that my conscience leads me to express the following conviction; that the child of Madame Gallois was not born *alive*, according to the meaning of our laws (n'a pas vecu, selon le vœu de la loi); for it did not breathe; and in fact the pulsations of the heart and of the umbilical cord exist (ont lieu) in the belly of the mother, and yet the infant does not enter into civil life, until it has breathed."

The same sentiments are expressed in the written opinion of Dr. Pelletan, as follow:—" In truth, neither the pulsations of the heart, nor those of the umbilical cord are sufficient to prove that an infant has *lived*, as the law requires, that is to say, has *breathed;* for these pulsations exist during intra-uterine life, and yet the child is not deemed *to be alive*, according to the meaning of the law.

The simple affirmation of M. Cabaret, the surgeon, that he observed the chest of the infant to heave up once or twice, cannot be admitted as a sufficient proof that respiration had actually taken place; and then the state of the lungs found on dissection, seems to point to the same conclusion. On the whole, I am satisfied that the child in question 'n'a pas vecu de la vie civile.' "

Two other physicians, MM. Guilbert and Auvity, expressed their entire assent to these opinions of Dubois and Pelletan.

The report of the three Commissioners, MM. Roux, Marjolin, and Marc, is very elaborate, touching upon all the points at issue, and examining with great minuteness, the depositions of each witness, more particularly the statements of M. Cabaret.

The conclusions to which they arrived, are:

That the infant, extracted by the Cæsarian operation from the patient Madame Gallois, was between seven and eight months old; that it was not alive (n'a pas vecu); but that it was *viable*, or capable of supporting life, seeing that there was no malformation or disease in any part.—*Annales d'Hygiene publique.*

---

### CASES FROM THE CLINIQUE OF M. BOUILLAUD, WITH REMARKS.

*Pulmonary Emphysema.*

In the ward of St. Jean de Dieu, at La Charité Hospital, there was, a few weeks ago, an old man affected with a chronic bronchitis, complicated with pulmonary emphysema, and with induration of the valves of the heart. For many years he had been subject to a troublesome catarrh; and when admitted into the hospital, his case exhibited all those features, which would have in former days been said to indicate a case of genuine asthma, but which now—thanks to auscultation—are known to be connected with certain definite pathological states of the lungs and the central organs of circulation. If, in such a case, the physician was to limit his examination to the respiratory organs, he would unquestionably be led into error; he would suppose that the disease was simply pulmonary emphysema, from the almost general vaulting or expansion of the chest, the loud resonance of every part on percussion, and the presence of a snoring noise (bruit ronflant) blending itself, at intervals, with a feeble vesicular murmur.

Such a diagnosis, correct as far as it goes, would however indicate a part only of the actual disease present.

In truth, pulmonary emphysema is very rarely met with alone and unconnected with any other affection of the lungs, or of the heart. It is a very frequent complication and co-existing disease; but seldom one which is met with by itself.

In examining the precordial region in the present patient, we at once discovered indubitable signs of a lesion of the heart. Its impulse was perceptible over a wider space than in health, and its *bruits*, especially over the left cavities, were unusually rough and accompanied with a blowing sound. There was therefore every reason to suspect the existence of valvular disease of the heart, probably of the semi-lunar valves of the aorta, and of the mitral valve also.

The treatment of such a case does not admit of very active practice. The view of the judicious physician is rather to prevent, as far as he can, the aggravation of the symptoms, than vainly to strive to remove those already existing. The occasional detraction of small quantities of blood from the cardiac region, by means either of leeches or of the cupping-glasses, very generally mitigates the dyspnœa and precordial uneasiness.

If depletion is unadvisable, the formation of slight issues with the caustic potass—not larger than a shilling or half-crown piece, and only so deep as to affect the dermis—is often very useful.

As to internal remedies, the best are perhaps light vegetable bitters, with the addition of a few grains of carbonate of ammonia, and of some preparation of squills. More good will be effected by regulating the functions of the digestive organs, than by using any medicines which may be supposed to act more immediately on the organs of circulation and respiration.

*Bronchitis.*

The case at present in the hospital affords a good illustration of the good effects of M. Bouillaud's practice of bleeding *coup sur coup* in arresting inflammatory affections of the air-tubes. There were present all the physical and physiological signs of a very extensive capillary bronchitis, complicated with a peripneumonic engorgement of the base of both lungs. The disease had lasted nearly three weeks, at the date of the patient's admission: it was attributable to exposure to cold, while the surface was in a state of free perspiration. Bleeding from the arm to 14oz., and the application of the cupping-glasses, immediately afterwards, over the seat of the most active inflammation, were sufficient to arrest the progress of the disease.

(This case was an instance of the truth of a remark, which M. Bouillaud has frequently inculcated on his pupils, that inflammations of membranous tissues are more obstinate than those of parenchymatous parts.)

On the morrow, the sub-crepitant râle was scarcely perceptible, the respiratory murmur was much more pure, and the pulse had fallen from 100 to 84.

*Angina.*

It is well known that with death are often effaced a number of physical changes or lesions, the existence of which had been distinctly recognised during the life of the patient. Such facts indeed are not unfrequently appealed to in depreciation of pathological anatomy. The following case affords an example of the *fugacité* of certain organic diseases. An aged, and rather unhealthy man, was admitted into the hospital for a saturnine (arising from lead) affection; while there, he was seized with angina. The larynx and pharynx were evidently the seat of active inflammation : the only diagnostic symptom of the disease, which was not present, was an alteration of the voice. This peculiarity gave rise to the suspicion that the angina was of an œdematous character. As the patient's strength was very feeble, general bleeding was considered inadmissible. Twenty leeches were therefore applied to the throat; and even *cette faible perte de sang* had the effect of inducing such a state of syncope, that the patient never recovered from it, and he died in 36 hours afterwards.

On dissection, no traces of inflammation were discoverable either in the pharynx or larynx; and the only morbid appearances found were some sanguineous coagula or concretions, which filled the cavities of the heart. Were these coagula formed gradually during the syncope, which preceded the fatal issue? It seems very probable that they were. Another question may be asked, can we suppose that in the present case the angina was only apparent and illusory, because anatomy was silent as to its phenomena? This conjecture is quite inadmissible in the judgment of those, who examined the case during the life of the patient; and we should remember that it is not at all more wonderful that the phenomena of mucous inflammation should be as evanescent after death, as those of erysipelas or of any other dermoid phlegmasia.

*Articular Rheumatism.*

"The duration of acute rheumatism is," says our author, "certainly the thing of all others in the world the least *vouée à la fatalité*, and the most under the control of remedial treatment." M. Bouillaud has most satisfactorily proved that the medium length of this disease, when treated with bleedings *coup sur coup*, is usually only from one to two weeks, whereas formerly it used to be from six to seven weeks.*

A man, 35 years of age, after exposure to wet and cold, was seized with pain and swelling of the feet, knees, and shoulders. He was twice bled from the arm, and also cupped freely within the first 24 hours after admission into the hospital. On the following day, the pains had almost completely ceased; and the subsequent convalescence was so rapid, that at the end of a week the patient wished to be discharged.—*La Lançette Française.*

---

### Subnitrate of Bismuth in Gastralgia.

This metallic salt has often been very indiscriminately employed in gastric affections, and has therefore, as might be expected, frequently disappointed the expectations of medical men.

The disease, in which it is especially useful, is idiopathic gastralgia, unconnected with inflammatory action, or with any organic lesion of the stomach.

The following cases may serve to direct the physician in his employment of this remedy.

*Case* 1. A literary man, 32 years of age, of a weak delicate constitution, and subject to hæmoptysis, was seized in the month of February with a general exhaustion and powerless, complete loss of appetite, nausea, and most uneasy distention of the epigastrium. The bowels were constipated; there were frequent alternate chills and flushings of heat over the body; and the sleep was generally restless and disturbed.

Four grains of the subnitrate of bismuth, night and morning, were prescribed. The dose was quickly increased to upwards of a scruple in 24 hours. No alleviation however of the gastric symptoms was obtained. The sulphate of quinine was therefore substituted, and the diet was made more generous and nourishing. An amendment rapidly followed, and in the course of a short time the patient was restored to health.

(There is surely nothing remarkable in these details. The case was clearly one of common dyspepsia, and any of the tonic vegetable bitters would doubtless have benefited the symptoms.—There is no mention made of gastralgia existing at any time.—*Rev.*)

*Case* 2. A workman had been suffering for five or six months from frequently-recurring attacks of cardialgia, which had resisted the employment of a multitude of remedies. The epigastrium was tender upon pressure; and even upon any sharp succussion of the body, as during sneezing, coughing, and so forth, the patient was often seized with a severe burning crampy pain in the region of the stomach. It was found however that friction, continued for some time, on this part rather abated than aggravated the uneasiness. The chief distress was experienced after tak-

* We have repeatedly taken occasion to impugn the accuracy of this, as well as of other favourite doctrines of M. Bouillaud. Perhaps there is no disease more strictly *humoral*, or in other words more depending upon an abnormal state of the blood, than severe acute rheumatism.

The excessively fibrinous condition of this fluid is a remarkable feature of its pathology; and we feel satisfied that the main indication in the treatment of the disease is to correct this condition—an object rarely to be achieved in less than three or four weeks.—*Rev.*

ing food; for then the gastric cramps came on almost immediately, and were accompanied with a sense of burning, and of extreme distention.

This state of suffering continued for about ten or fifteen minutes; but was always renewed upon swallowing either solids or fluids. The case was regarded as one of severe gastralgia. All food was debarred, and the patient was enjoined to quench his thirst by sucking small pieces of ice. The use of the subnitrate of bismuth was also commenced, and continued for several days; but the stomach pains seemed rather to increase than to abate under this treatment. Warm bathing and minute doses of opiates were substituted, and under this regimen the attacks of pain were gradually suspended.

*Remarks.* In the preceding case, the subnitrate seemed to act as a direct irritant, and to increase the very symptom for the relief of which it was administered.

Indeed this salt appears to be not so well suited for those cases in which there is an extreme sensibility of the stomach, as when this viscus is subject to attacks of irregular crampy convulsion.

Whenever the paroxysms of gastralgia become excessively acute, it is necessary to have recourse to opium. The subnitrate of bismuth is most serviceable when the gastralgia is chiefly of a spasmodic character, and the pain is not the most prominent, but one only of the secondary or super-added symptoms.

*Case* 3. Madame B., 42 years of age, whose nervous system had suffered much from severe chagrin and distress, had experienced four years ago a serious illness, the chief symptoms of which were a partial paraplegia, numbness and intermittent pains in the limbs, and a constant (aggravated however at intervals) state of delirious confusion of mind. Occasionally the patient recovered all her mental tranquillity and intelligence for a short time; but this state was soon succeeded by one of bewilderment and raving.

All these unpleasant symptoms were removed under the use of musk, camphor, and quinine.

A few months ago, Mad. B., after meeting with a troublesome wound, was seized with a shivering fit, accompanied with cramps in the limbs, excitation of the cerebrum, and with a most acute constrictive pain in the epigastric region.

M. Recamier was called into consultation. He quite agreed with the physician in attendance, that there was no organic disease existing, and attributed all the symptoms to a nervous disorder of the stomach. Mild antispasmodics, conjoined with the subnitrate of bismuth, were administered; and by persevering in the use of these remedies for a few days, the patient was entirely relieved from all her sufferings.

Before concluding these remarks, we may state that in several cases we have observed excellent effects from a combination of opium with the subnitrate of bismuth—the dose of each varying according to the predominance of nervous pain, or of muscular cramp.

In some instances too, the quinine may be very advantageously associated with the metallic salt.—*Bulletin General de Therapeutique.*

---

### Practical Observations from the German Journals.

### *Hooping Cough.*

Dr. Waldeck of Berlin has been very successful in abridging the duration of this troublesome complaint by means of small doses of belladonna. Its use may be commenced with a twelfth or tenth part of a grain. Whenever any febrile excitement of the system is induced, the medicine should be at once discontinued.

Dr. Joel strongly recommends the following formula, when the disease has reached the *stadium nervosum,* unattended with any complications, and when neither depletion, nor the use of emetics is judged advisable.

℞. Extract. pulsat. nigric.

Sulph. aurat. antimon. āā gr. iij.

Extract. Hyosciami, gr. vj.
Sacchar. lact.
Pulv. Gummi, āā ℈j.
M. in pulv. xij. divide.

One of these powders is to be given every four or six hours.

*Ulcers on the Nipples, &c.*

Dr. Boehm of Berlin has found great benefit from the employment of a desiccative powder,—composed of one part of oxyde of zinc and one or two parts of powdered gum arabic — sprinkled upon these troublesome little sores. It forms a crust or thin cake over the ulcerated surface, which, being thus protected from all irritation, speedily heals.

*Treatment of Thymic Asthma.*

Dr. Hachman has contributed a very valuable paper on the history of this disease of infants to a late number of the *Hamburger Zeitschrift.*

He regards it as a purely spasmodic or nervous affection : and in its treatment avoids all very active and violent measures. In the first stage, the doctor recommends a powder composed of oxyde of zinc, ipecacuan, and the extract of the lactuca virosa, three or four times in the course of the twenty-four hours. To stout robust children he usually administers calomel at the same time.

When the digestive powers are weak, small doses of rhubarb and the carbonate of soda will be found useful.

If the patient be of a scrofulous habit, some preparation of steel, and a residence in a pure bracing atmosphere, will greatly facilitate the cure.

*Chronic Vomiting.*

A man, 58 years of age, had been distressed for upwards of two years with vomiting after every meal of food. The epigastric region was quite flaccid, and not at all tender upon pressure. Powders composed of camphor, cayenne pepper, charcoal, and canella, along with frictions over the stomach with turpentine, ammonia, and tincture of cantharides, effected a complete cure.

A man, 50 years of age, had been subject for several years to vomiting, accompanied with pain in the epigastrium, the region of which was very hard to the touch, and exceedingly distended. The patient had become greatly emaciated.

The use of lime-water and extract of hemlock ultimately dissipated every unpleasant symptom.

A man, 58 years of age, had for a great length of time been distressed with frequent return of vomiting : there was no pain, but most annoying flatulence and obstinate constipation. He was ordered an aromatic bitter infusion, and minute doses of aloes. Under this treatment he quite recovered.

*Nitrate of Silver in Gastralgia.*

Dr. Steinitz has found this metallic salt successful in some severe and obstinate cases of this disease, which had resisted all the ordinary remedies, such as musk, castor, morphia, hydrocyanic acid, the preparations of zinc, bismuth, &c.

It may be stated, as a remark of very general truth, that metallic salts have a very marked effect upon all nervous affections, attended with high sensibility.

The excellent effects of the oxyde and sulphate of zinc in chorea, of steel in many cases of neuralgia, cupreous salts in spasmodic croup, &c. all tend to prove the correctness of this observation.*

---

### CASES OF MALIGNANT PUSTULE IN MAN.

Several cattle on one farm died in the second week of last July, rather suddenly, and without the cause of their

---

* Our German friends seem to be not aware that the internal use of the nitrate of silver in certain cases of gastric suffering was first recommended by Dr. Johnson, the senior Editor of this Review.

It is an admirable remedy in allaying the morbid sensibility of the stomach in some forms of Dyspepsia.—*Rev.*

death being found out. The peasants in the neighbourhood, being either unaware of, or disregarding the fact, partook freely of the meat. A few days subsequently, most of these people were taken ill with flying pains through the bowels, vertigo, general lassitude, &c. Two men, who had assisted in skinning and cutting up the carcases, were most seriously affected with feverish symptoms.

More of the cattle having died, attention was now paid to the state of their bodies.

At several places, especially around the neck, there were observed numerous subcutaneous watery tumors; and on opening the abdomen, the most remarkable appearance was the softened gangrenous state of the spleen, which viscus seemed to consist of a membranous bag, filled with a thick and dark-coloured blood.

No doubt could now exist as to the genuine *carbuncular* nature of the disease. M. Wagner (the author of these observations) gives it the name of gangrene of the spleen (milz-brand), from its most remarkable pathological character.

One of the two men, who were most severely affected, died on the 20th of the month, although he had, with great effort indeed, been able to go out on the preceding day. The symptoms during the last twenty-four hours were excessive prostration, intense vomiting, coldness of the limbs and body, bloody diarrhœa, convulsions of the extremities —in short, most of the phenomena of aggravated cholera.

On the same day (20th) a woman, who had eaten of the meat, was affected with similar symptoms and died on the following day. A dark gangrenous-looking pustule was found on one of the thighs. In little more than twenty-four hours after death, the bodies of these persons were so thoroughly decomposed, that they were almost fluid; and no examination therefore could well take place.

On the 22nd and 23rd, nine other persons were affected in a similar, though less severe, manner. In all, the morbid phenomena were nearly alike: the skin was dry, and the pulse small, feverish, and sometimes scarcely perceptible; there was a sense of an oppressive anxiety about the precordia, with nausea and tendency to vomiting. In most, carbuncles or anthorax-like tumors on different parts of the extremities and body were present; and although these were usually small, there was often experienced over a large space a feeling of great pain and intense burning.

The treatment pursued by M. Wagner was as follows. When there were no carbuncles, he contented himself by ordering poultices of linseed meal, bran, and vinegar to the epigastrium, the use of acid drinks, and a light spare diet. When however there were any carbuncles, the treatment was much more active. He made a crucial incision through each tumor, and cauterised it deeply with the caustic potash: the cauterisation was scarcely felt. The part was then covered with linseed poultices, and with powdered charcoal mixed with vinegar.

Internally he administered small doses of camphor, and a strong decoction of cinchona with the anodyne mineral drops of Hoffman.

All these patients, with the exception of one, an old woman who had a carbuncle on the thumb, were better on the following day.

In her the symptoms became more aggravated, although the tumor had been incised and well rubbed with the caustic. The whole arm was swollen and inflamed up to the shoulder; the fore-arm was covered with livid phlyctenæ; and these local symptoms were accompanied with fever, diarrhœa and extreme prostration of strength.

M. Wagner ordered a cataplasm of cream cheese (cataplasme de fromage à la creme) to the skin. Quite contrary to expectation, this old woman ultimately recovered, although the eschar was long of falling off and the sore was very tardy in healing.

Eight days after the seizure of the last mentioned patients, two other persons were taken ill: they, it seems, had not eaten of the meat; but they had handled the carcases of the dead bullocks. In both, some carbuncles made their appearance along the arms.

Ten days later, another fatal case of infection manifested itself. It occurred in one of the servants of the farm, to which the diseased cattle belonged. He had not only handled, but had also eaten of, the corrupted meat. Nevertheless he continued quite free from any symptoms of ill-health, for a full fortnight after the first breaking out of the disease. At length however he became affected with the usual train of phenomena, and, in addition to the constitutional disturbance, a large carbuncle appeared on the left fore-arm. On the second day he died.

M. Wagner made the following experiment. He melted some of the fat of one of the diseased bullocks, and gave it to two guinea-pigs, two dogs, and two cats. Every one of these animals died.

From his enquiries he is led to conclude that the carbuncular fever, or gangrenous splenitis, whether it be attended with pustules or not, is in all cases propagated either by the ingestion of the diseased meat, or by cutaneous absorption from handling it, and never by merely atmospheric miasms. Emetics in the first stage are generally useful, and afterwards cordial diaphoretic stimulants may be given with much advantage.—*Hufeland's Journal.*

---

### On the pernicious Effects of certain Cosmetics.

The following cases will serve, in some degree, to prove what deleterious effects are sometimes produced by these much-vaunted remedies.

A gentleman, who deemed himself quite unfortunate from having red hair, being anxious to beautify himself by dying it black, had recourse to a preparation, which he had noticed was most highly recommended by some of the journals. The effect of the application was to induce an attack of erysipelas on the part, so severe that he was under medical treatment for a considerable time.

The dupe was determined to prosecute the tradesman; and, on the trial, MM. Chevalier and Marc were summoned to declare the composition of the cosmetic, which had been sold. It was found to consist of 30 parts of oxyde of calcium, 2½ parts of oxyde of lead, and a minute trace of silica.

A gentleman had consulted his physician in consequence of troublesome colic and constipation, to which he had been subject for some time. Certain circumstances led the physician to suspect that the abdominal symptoms might possibly be caused by a pommade, which his patient had been long in the habit of applying to his hands, for the purpose of making them delicately white! He requested M. Chevalier to analyze it. It was found to consist of 5 parts of cerate, and 3 of white carbonate of lead. The mere discontinuance of this cosmetic was sufficient to remove every unpleasant symptom.

Madamoiselle G. having read in one of the newspapers of the extraordinary effects of a certain application, Eau de Perse, in dying the hair of a black colour, purchased a phial of it for use. She was however much surprised to find that not only did those parts, to which the fluid was applied, became blackened, but also that various parts of her person acquired a similar discolouration.* M. Chevalier recommended to use first a solution of chloruret of sodium, and then one of ammonia, as a wash. The Eau de Perse was found upon analysis to consist of ten grains of nitrate of silver in an ounce of aromatic distilled water.

The fourth case was very similar to this one; but the symptoms were much more severe, in consequence of the

---

* Surely this must have been mere fancy; unless indeed some of the caustic liquor had been accidentally dropped upon the discoloured parts.

We cannot well believe that the application of the nitrate of silver to one part of the body should at all affect a remote part. The internal use of this salt is a very different thing; as, under such circumstances, it is no doubt absorbed into the system.

much greater strength of the caustic solution.

The tradesman, who had vended the cosmetic, was prosecuted by the King's Advocate, and *condamné* by the court.—*La Lançette Française.*

*Remark.* Most of our readers are probably aware that all the preparations sold to blacken the hair contain more or less of the nitrate of silver.

---

### On the late Prevalence of Variola in London and in Paris.

Our metropolitan readers are no doubt well aware that, during the last six or seven months, there has been an unusual prevalence of the variolous contagion in and around London; and the same has been found to be the case, we believe, in numerous districts of the country.

From a short article in a recent number of one of the French medical journals, it seems that small-pox has existed to a very considerable extent in Paris also for some months past.

In almost every hospital numerous cases were observed; and in some of the districts or *arrondissements* of the metropolis, it appears to have been unusually fatal:—in one alone there was twenty-three deaths in the course of a month.

The disease was not confined to any particular age: but old people certainly were very rarely affected with the contagion.

Many of the patients had either been vaccinated, or had passed through the small-pox (we suppose from inoculation).

In not a few instances was the disease of a confluent character; and some cases proved very rapidly fatal from the affection of the throat and chest.

The precursory symptoms were very generally severe pains in the limbs, alternations of chills and flushings of heat, intense headache, and irritability of the stomach.

The epigastric pain however—so much insisted upon by Sydenham as one of the most pathognomonic premonitory signs of variola—was very frequently absent.

The eruption usually made it appearance on the second or third day; often it did not cease at this period, but continued with much severity, and became now complicated with a most troublesome angina. The inflammation of the throat and the congestion of the lungs were certainly the most troublesome and dangerous symptoms; and hence when these were at their *acmé*, the risk was the greatest.

In all the fortunate, although severe, cases, the pustules filled well, the face was enormously swollen, the fever was high, but the respiration, however uneasy, was not seriously distressed. On the other hand, in the fatal cases, the pustules remained low and imperfectly distended, the swelling of the features was often not great; but the breathing was much quickened from the beginning, and became more and more difficult, as the disease advanced.

A few cases proved very troublesome after the dessiccation of the pustules had taken place, in consequence of numerous boils and circumscribed abscesses making their appearance on different parts of the limbs and body.

With respect to the treatment which was found to be most useful in this epidemic of variola, the cautious employment of blood-letting in the very early stage of the disease was certainly beneficial. It seemed to facilitate the eruption of the pustules, and also to promote their maturation.

The use of the lancet however in variola always requires much discretion, as we must never forget what expenditure, so to speak, of vital energy Nature has to undergo in the subsequent stages of the disease. When we are at all doubtful as to its employment, it is better to trust to the administration of emetics and nauseants to reduce any excessive pyrexia, which may be present at the beginning of the disease.

When the eruption has fairly taken place, and provided there are no unpleasant local complications present, the best therapeutic rule is *laisser faire la Nature.*

The most critical period or stage of the disease is that of the suppuration of the pustules: the danger arising either from a deficiency of vital energy, or from a feverish excitement—often connected with some local mischief in the throat, lungs, &c. of the system. Certainly one of the most useful of remedies in this stage of variola is the application of blisters to the lower extremities—either to the thighs or to the legs. The exhibition of gentle aperients, with mild vegetable tonics, is generally useful towards the close of the disease. —*Bulletin Gen. de Therapeutique.*

---

Hysteria, singular Case of, in which there was a Discharge of the Urine from the Umbilicus.

A young female, sixteen years of age was admitted in October 1836, into the hospital, to be treated for an affection of the spinal marrow.

She complained of headache, vertigo, great wakefulness, and extreme muscular weakness. The spinal column was tender on pressure at some points: and the scars of old issues were visible over the lumbar part of the back. She had a troublesome tickling cough, and shortness of breath upon any exertion. The abdomen was knotty, tympanitic, and tender; the umbilicus was much depressed, forming a sort of *cul de sac*, about an inch deep, from which exsuded a fluid, which stained the linen of a reddish colour, and left a thin coagulum or crust on drying. A similar fluid was discharged from the ears. No urine had been voided for thirty-six hours previous to her admission into the hospital; and only five or six ounces escaped by the catheter. The catamenia had been regular.

It appeared from the girl's report that, in the Spring of 1836, she had been seized with erratic pains in various parts of the body, and at length with a fixed uneasiness in the spine, accompanied with pulsations in the throat, and great difficulty of breathing. The bowels were exceedingly constipated; and sometimes three or four days passed without more than a few ounces of urine being voided. A reddish-coloured fluid exsuded from the umbilicus, and from both ears: it had a distinctly urinous odour.

This state of things continued for some months. In the following August, she began to vomit a saltish, urine-smelling fluid; and on examination it proved to be urine. This strange phenomenon recurred several times, when there was a suppression of the secretion in the natural way.

In the following month, September, the exsudation from the umbilicus returned; and it continued up to the period of the report.

The catheter was ordered to be introduced every day; but very little urine was ever drawn off: it was always very high-coloured and even bloody. The urinary discharge from the umbilicus and also from the ears—amounting to about an ounce daily—continued regularly till the middle of December. During all this time, the girl suffered much from head-ache, vertigo, severe pains in the loins, paroxysms of dyspnœa, distention of the abdomen, &c. The catamenia were however regular.

Since the umbilicus ceased to exsude any fluid, the abdomen became much less tender.

In the following March, the urinous vomitings recommenced;—from 20 to 40 ounces were rejected in the course of the twenty-four hours. The results of a chemical analysis left no room for any doubt as to the genuine nature of the rejected fluid.

In the month of June, she left the hospital, improved in health in many respects; the urinous vomitings had in a great measure ceased, and the secretion had returned to its natural channels.

The seat of the spinal affection was over the 11th and 12th dorsal vertebræ. —*La Lançette Française.*

We believe that the preceding report has been transferred from one of the British medical journals,—probably the Edinburgh one—to the pages of our Continental cotemporary.

It had escaped our notice in the original. The reporter is Mr. Laycock.—(*Rev.*)

---

### On the Therapeutic Uses of Strychnine.

The following observations are from the pen of M. Bally, now one of the physicians (formerly of the Hôtel Dieu, where his clinique used to be attended by a large number of students) of La Charité Hospital, and will therefore be received with deference.

Strychnine is an alkaloid, which is obtained from the *strychnos nux vomica*, and the *strychnos santi ignatii*—both of which belong to the natural order of the *apocyneæ*. The latter of these plants is found to contain three times as much of the genuine alkaloid as the former; another alkaloid, however, the *brucine*, exists in larger quantity in the nux vomica than in the strychnos sancti ignatii.

M. Magendie had discovered, by the results of his experiments, that strychnine has a special or specific action on the spinal marrow. Such being the case, it was reasonable to predict that the use of it would be attended with numerous difficulties in all cases of cerebral hæmorrhage, and consequently in those forms of paralysis, which have their origin in such a cause. M. Bally speedily found the truth of this remark in practice.

Although the palsied muscles were affected with twitchings, and, as it were, electrical movements, no permanent advantage was ever obtained in such cases; and in not a few instances the cephalic symptoms were decidedly aggravated. Vertigo, noises in the ears, sleeplessness, and suffusion of the features—all indicated a manifest threatening of further mischief.

But such were not the effects in those palsies, which were dependent upon uncomplicated lesions of the spinal marrow. In an immense number of such cases the benefit, derived from the use of the strychnine, was decided and permanent.

Those paraplegiæ, whether partial or complete, which are not dependent upon a deep-seated organic lesion—such as the actual separation of the nervous bundles of the medulla in consequence of *ramollissement*, a compression from a displacement of any of the vertebræ, or from the presence of any foreign body in the spinal canal, &c.—derive the most positive relief from the use of the strychnine.

*Action of the Strychnine.* We have already alluded to the marked influence of this most potent drug in determining a fulness of the cephalic vessels. It is of the greatest importance to attend to this circumstance, as we are satisfied from the results of an extended experience that its use is always attended with much risk in all diseases of the cerebrum.

Some practitioners have recommended the employment (internal) of strychnine in cases of amaurosis. M. Bally's experience does not confirm the justness of this praise. The external or endermic use of the alkaloid is more frequently successful in paralytic affections of the eye. As a matter of course, we cannot expect any advantage when the amaurosis is the result of any serious lesion of the retina, optic nerve, &c. Strychnine does not seem to exert any marked effect on the organs of digestion: there may be a certain degree of excitation follow its administration, but this, along with an intense bitter taste in the throat, is perhaps the only one.

Perhaps this is to be attributed to the very rapid absorption of the strychnine, and to the circumstance too of its action being altogether *specific* or *elective*.

If it sometimes acts on the urinary bladder, so as to remove paralytic weakness of this viscus, it is not by a direct, but by a *reflected* influence. The spinal marrow is in the first place excited, and this excitation is transmitted to the lumbar and sacral plexuses, and thence to the nerves of the pelvic viscera.

In the same manner are the stimulant effects of strychnine on the nerves of the upper and lower extremities to be explained.

One of the most decided signs of the agency of strychnine in such cases is the occurrence of partial convulsions, or crampy twitchings of certain muscles. If its use be incautiously persevered in after the manifestation of these phenomena, a state of complete tetanus may be induced.

M. Bally says that, in some cases, the twitchings or jerkings of the muscles have, in his practice, been so powerful that he has been obliged to have the patient tied down upon the bed, as it required two attendants constantly to restrain the violence of the *boundings*.

Fortunately these effects do not usually continue long: they commence soon after the ingestion of the medicine. It is of importance to be aware of these facts.

Few physicians have ever administered the strychnine against saturnine or lead colic; but it has been used with efficacy against those partial palsies of the limbs, which not unfrequently follow this disease. M. Bally however has in numerous cases of the primary affection had recourse to this potent alkaloid, when purgatives have failed in mitigating the symptoms.

In such cases he has been in the habit of combining the strychnine with the muriate of morphia—a grain of the former and half a grain of the latter made into 16 pills—from two to four or six to be given daily.

As a general rule for the internal administration of strychnine, we may state that we should commence with from a twentieth to a sixteenth part of a grain for a dose; and this quantity should not be increased more quickly than every six or seven days. It is rare that any patient can bear, even after a protracted use of the medicine, more than two or three grains in the course of twenty-four hours.—*Bulletin General de Therapeutique.*

---

### Results of Re-vaccination in Silesia.

Dr. Aggens practised re-vaccination on 962 persons, during the year 1836. The operation succeeded perfectly in 822 of these cases: in 68, the pustules did not exhibit a mature or complete development; and in the remaining 72, no effect whatever was produced by the punctures.

In many cases, the regular and characteristic vaccine pustule made its appearance as early as the third, and had acquired its full size by the fifth or sixth day. In general, the author remarked a more decided reaction in the lymphatic system, than usually happens after a first vaccination. Often did the patient, in the course of two or three hours after the operation, begin to experience a smarting sensation along the arm and in the armpit of the affected side. During the period of suppuration, the arm not unfrequently swelled considerably, and the system became somewhat feverish. But the pustules resulting from re-vaccination never exhibited *cette belle coloration perlée* of those produced by the first operation: they were of a greyish hue. On the whole too, their progress was decidedly more rapid, more especially in their last stage, or that of drying and desquamation. In some cases, in which no regular or characteristic pustules were formed, the punctures became nevertheless surrounded with an areola of inflammatory redness.—*Pfaffs Mitheilungen.*

---

### Results of Re-vaccination in the Prussian Army, during 1836.

The entire number of persons re-vaccinated was 42,124. Of these, 32,635, exhibited the cicatrices of a former operation on their arms. In 6,543 persons, the cicatrices were unsatisfactory; and in 2,840 there were no traces at all of them.

In the re-vaccinations of this year, regular vaccine pustules or vesicles were formed in 18,136 instances; in 9,940, they were of irregular or imperfect developement.

In 14,048 the operation failed in producing any effect; but on repeating it a second time, it took effect in 1,569 cases, and again failed in 8,205 cases.

The number of pustules induced varied from one to thirty in different individuals.

Among all the persons re-vaccinated during this and the preceding one or two years, 14 only were attacked with any of the forms of the prevalent varioloid disease: there was not, however, a single example of genuine small-pox met with, although the epidemic existed in the country.

The lymph employed was very generally taken from the arms of young children; and lymph that had become dry was always avoided.*

On comparing the results of re-vaccination during 1836, with those of preceding years, the reporter states that he is led to the conclusion that the disposition or tendency to contract a new cow-pox, (in other words, to be affected by re-vaccination), and consequently the liability to variola, seem to be increasing every year. Of 42,124 persons re-vaccinated in 1836, the operation succeeded perfectly in more than 18,000; whereas of 48,478 persons re-vaccinated in 1833, only 15,269 were affected; of 44,454 in 1834, only 16,679 were affected; and of 39,192 in 1835, only 15,315 were affected.—*Medicinishe Zeitung.*

* Attention to these two particulars should be always paid in practising re-vaccination. The lymph should be taken from the arm of an infant, and it should be inserted before it has become dry. Our own experience had led us to the same conclusion, before we read this German communication. —(*Rev.*)

### Treatment of Dropsy of the Bursæ Mucosæ, with Iodine.

This practice has of late years been followed with great success in the large Marine Hospital at Toulon. Hygroma, or dropsy of the mucous bursæ adjacent to some of the large joints, is of frequent occurrence among men who are engaged in fatiguing and laborious occupations.

The knee, and more especially the left one, is the joint most frequently affected. Perhaps the most common cause of the complaint is some slight external injury of the part, such as a blow, a fall, or long-continued pressure. The swelling usually appears at first over the centre of the patella, thence gradually extends on all sides, and, if permitted to increase, entirely covers at length the whole front of the joint.

It is only when the tumor has attained a very large size that the affection can be mistaken for *hydarthrosis,* or genuine dropsy of the articular cavity.

Hygroma consists in a distention of the bursæ mucosæ, either with a simple serosity, or, more rarely, with a sanguinolent fluid. When the swelling follows a contusion, its development is rapid, and the fluid, contained within, is usually more or less decidedly bloody: but this is not the most common progress of the disease. If the swelling has continued for a considerable time, the contents of the bursæ become more consistent, and in some cases almost semi-solid, with flocculi of albumen through it. The parietes also of the bag are apt, under these circumstances, to grow thicker and more firm and resisting.

M. Boyer, in treating of hygroma, says that he has almost always found the swelling, when recent, readily dispersed by the topical application of a solution of the muriate of ammonia—an ounce of the salt to a pint of water. The resolution is however generally very tardy.

The experience of other surgeons quite bears out the truth of the last statement. If the tumor has continued for any considerable length of time, the use of the muriate, as recommended by Boyer, will not suffice.

Various modes of treatment have been suggested in chronic cases; compression, incision, excision of part or whole of the cyst, puncture either alone, or followed by injection of the sac, &c., have been recommended by different writers. The frequency of the complaint in seamen, carpenters, &c. has enabled M. Reynaud, the chief surgeon of the naval hospital at Toulon, to test the comparative value of various remedies.

He quite rejects the employment of mere unassisted compression. The treatment by making an incision into the sac, although more efficacious than that by compression, is however so objectionable that he at once discards it; and as for the excision or complete removal of the swelling, no surgeon would ever think of so severe a remedy, unless every other means had failed, and the parietes of the cyst had become exceedingly thickened and indurated.

Puncture, if followed by appropriate compression, is decidedly preferable to any of the preceding methods of cure.

It may be very properly had recourse to in all cases, where the use of iodine and of other resolvents has failed; but the surgeon should not premise a permanent cure even from puncture; as the effusion is apt to return, and a second operation will then be required.

M. Reynaud very properly condemns the practice of injecting a stimulant fluid into a bursal sac under any circumstances.

On the whole, the simplest, and in most cases, the most efficacious, remedy is friction with an ointment of the hydriodate of potassa—two drachms to an ounce of lard. This is to be used morning and evening; and after each friction, the joint should be covered with a linseed poultice.

Some few trials with the *ioduret of lead* seem to indicate that this preparation is more active that the *hydriodate of potassa*.

The average time required to dissipate the swelling is a fortnight or so.

The reporter adduces numerous cases in proof of the efficacy of the treatment now recommended. Several of these however were of very short standing; and hence we (the Rev.) do not attach much value to them, as it is well known that synovial swellings will often dissipate of themselves, when no remedies are employed. The most chronic case was one of six months' duration: the tumor had acquired the size of the fist; but it was speedily dissipated by frictions with the iodine ointment.—*Bulletin General.*

*Remark.*—In old-standing cases, unaccompanied with pain, we have repeatedly succeeded in causing the bursal swelling very quickly to disappear by striking it smartly with a book—till its parietes seemed to have given way—and then covering it with mercurial plaster spread upon leather.—*Rev.*

---

### Dropsy of the Joints successfully treated with Emetics.

The following cases occurred in the hospital of the Hôtel des Invalides under the care of M. Gimelle, who has the merit of having first established the efficacy of the treatment of articular dropsies by large doses of the tartrate of antimony, and who intends shortly to publish a treatise on the subject.

*Case* 1.—A soldier, 36 years of age, still suffering from the dregs of syphilis, had been seized with pains in his right knee, a fortnight after his admission into the hospital. The joint became swollen, hot, and excessively tender. In the course of a few days the sense of fluctuation was quite evident. For three weeks the case was treated with leeches, blisters, &c. and with antiphlogistic and emollient medicines; but the relief, thus obtained, was only very partial.

At this period the antimonial treatment was commenced; it was continued for eleven successive days, according to the following method. On the first day 4 grains were given; and this dose was daily raised by 2 grains each day, till the patient could *tolerate* 12 grains—which quantity was persevered in for three days.

It was observed that whenever the *toleration* of the medicine was induced (on the fourth day) the local affection of the knee began rapidly to subside. The cure was complete at the end of a fortnight. But now unfortunately the left knee began to be similarly attacked; a similar plan of treatment was adopted, and with equal success.

*Case* 2.—This patient too was in the hospital for secondary syphilis. During his stay, he was affected with an inflammatory attack of the right elbow joint. For the first ten days the case was treated in the usual method, with leeches, poultices, blisters, friction with iodine ointment, &c.; but without very decided relief. The antimonial medication was therefore substituted; and in the course of nine days all symptoms of the arthritis were dissipated. The daily dose had been gradually raised from four to 12 grains.

*Case* 3.—A soldier was received into the hospital of the Hôtel for a dropsical affection of one of his knee-joints.

The swelling was very great, but it was not accompanied with much pain. The fluctuation was quite distinct. The joint was freely leeched, and poulticed, and subsequently blistered; but it was not until the antimonial treatment was fairly commenced that any decided amendment was visible. From this period, the patient rapidly improved in every respect.

In the remarks, appended to the reports of the preceding three cases, we are informed that the antimonial treatment has been successfully used by M. Gimelle in upwards of twenty cases of articular dropsy; and that it is applicable equally to chronic and to acute cases of the disease. He has never raised the dose of the tartrate above 16 grains in the 24 hours; nor has he ever continued its administration beyond 17 or 18 days.

It was always much more speedily and much more certainly efficacious, when it (the tartrate) was well *tolerated*, *i. e.* did not produce much vomiting or purging. In most cases these disagreeable effects continue for two or three days; but then they subside and are followed by profuse perspirations and ptyalism. Occasionally the treatment seemed to cause a feverish excitement of the whole system for a short time.—*La Lancette Française.*

### M. Velpeau on the Treatment of Blepharitis.

M. Velpeau recognises four species of the disease—the mucous, the glandular, the granular, and lastly that form, which occurs in new-born infants.

### 1. *Mucous Blepharitis.*

Decidedly the best remedies for this form of the disease are local astringents. Of these the nitrate of silver, the sulphate of zinc, and the sulphate of copper in collyria are to be preferred to all others. On the whole, the first of these is the most efficacious, and trustworthy; and therefore of late years M. Velpeau has had recourse to it in almost all cases. He usually commences its use by ordering from a half to an entire grain to one ounce of distilled water: the dose is to be gradually increased to six, or even ten grains.

A few drops of the solution are to be dropped into the affected eye, two or three times in the course of the day. The finger should then be pressed upon the eye-ball, and the patient bid to move it about freely, so that the solution reach every part. This practice is to be repeated regularly for four or five days; and it may then be omitted for two days or so, before it is resumed. It is usually during the interval of repose that the amendment is most obvious. By persevering in the treatment now recommended, for two or three weeks, a cure may very generally be effected.

Whenever there is headache, flushing of the face, or any symptoms of fulness of blood about the head or eye, the use of antiphlogistic measures is, as a matter of course, to be resorted to.

### 2. *Glandular Blepharitis.*

In this form, astringent pommades are to be preferred to collyria; as the former remain longer in contact with the diseased surface than the latter. The *Pommade de Janin*, (this contains, we believe, the red oxide of mercury, *Rev.*) or an ointment prepared with the white precipitate, or with the nitrate of silver, is perhaps the most efficacious formula, that can be adopted. It is not however indifferent which ointment is to be used in each case. M. Velpeau says that, from multiplied experience, he has been led to the following conclusions respecting their comparative efficacy.

If the glandular blepharitis assumes at all the *diphtheritic* form, he prefers the *white precipitate* ointment (ʒj. to ℥j. of lard); if there be excoriations on the free edges of the eye-lids, the ointment of the nitras argenti is the best; and lastly, when the excoriations are more considerable and deeper than usual, the use of the solid nitrate, applied directly to the part, is more efficacious than any ointment.

In the treatment of this form of blepharitis, we should never expect a very rapid cure: it is almost always very obstinate and tedious, whatever treatment is adopted. Hence the prudent surgeon will be cautious in not promising a very speedy amendment.

3. *Granular Blepharitis.*

This is unquestionably the most perverse and intractable form of the disease.

Collyria, of all kinds and degrees of strength, are generally of no decided or permanent utility; and hence they are never to be trusted to. The various eye-ointments, which are so useful in many ophthalmic diseases, are in very many cases equally inefficacious.

In 1831, (M. Velpeau) began to use the solid nitrate of silver as a local application to the diseased surface. The first case was a very obstinate one, having lasted for several months at the date when it came under my care. The upper eye-lid was chiefly affected; its conjunctival lining was much thickened and covered with fungosities and granulations.

I applied a stick of the nitrate of silver lightly over the diseased membrane: the application was renewed several times after the interval of five or six days. The cure in this instance was complete.

But let it not be supposed that this treatment will prove equally efficacious in all instances. M. Velpeau confesses that, often in his own practice, it has quite failed of giving permanent relief. The sulphates of copper and of iron may be used in the same manner; and in some instances they have been found of marked utility, when the nitrate had failed.

4. *Blepharitis of New-born Infants.*

This form of the disease is perhaps the most serious and alarming of all. In some cases the destruction of the tissues of the eye is so rapid in its progress, that no remedial means have any power in arresting it. Generally however an appropriate treatment, provided it is instituted at an early stage of the disease, will speedily effect a cure.

On the whole, we should trust rather to topical, than to constitutional remedies. The latter indeed are not to be neglected; but, *alone*, they will not suffice.

The most potent of all local remedies is unquestionably the nitrate of silver, either in solution, or, what is usually more efficacious, applied in the solid form to the inflamed mucous membrane.—*L'Experience.*

---

### Case of Phlebitis after Venæsection, with Remarks by M. Blandin.

A young girl, 18 years of age, was admitted into the Hôtel Dieu for chlorosis and amenorrhœa. Symptoms of pelvic irritation having come on, she was bled from the arm. On the third day after the venæsection, she began to complain of uneasiness and pain in the seat of the puncture, which was found to be gaping, with its edges somewhat everted. When pressure was made along the line of the cephalic vein, the patient felt pain, and the vessel was hard and tense, for about two inches upwards, to the touch.

Thirty leeches were immediately applied to the seat of the injury; but the tension and uneasiness along the *trajet* of the vein were rather increased than abated towards the evening, and the wound now discharged a purulent matter: forty leeches more were therefore ordered.

On the following day, the whole arm was exquisitely painful, and even the fore-arm began to be tender and swollen, more especially along the course of the veins. In the evening, the patient had a decided shivering fit,—indicating the commencement of a purulent infection of the system. The arm was again very freely leeched, and the patient confined to refrigerants and low diet. Next day the arm itself was rather easier; but the axilla and fore-arm were excessively painful, and even the palmar veins seemed to be involved in the inflammatory disease: the hand was œdematous and somewhat benumbed. Thirty leeches were applied, night and morning, over the parts of the extremity, which were most sensitive.

At this period, M. Blandin was requested to see the patient, who had been hitherto under the care of M. Husson, one of the physicians of the hospital. The same mode of treatment was persevered in; other forty leeches being ordered to be applied. Fortunately this was the last time that any more depletion was found necessary, as the symptoms now began to abate. In all, 250 leeches were applied, in the course of between four and five days, to a patient, who was previously in a state of great debility. The extremity remained still much swollen from œdema; and, what was much more serious, symptoms of pleurisy and of pleuritic effusion on the right side began to manifest themselves.

Notwithstanding the preceding illness, the patient was bled twice from the arm, and a number of leeches was applied over the seat of the thoracic pain, with the happy result of checking the disease. The patient remained for a length of time exceedingly reduced; but ultimately she quite recovered her general health and the free use of the affected arm.

M. Blandin, in his comments on the preceding case, alludes to the apprehensions which he had of its issue, especially when the symptoms of pleurisy made their appearance. Every pathologist knows that whenever the system becomes infected with purulent absorption, there is a remarkable tendency to local inflammations in some of the internal viscera, and more frequently in the lungs and liver than in perhaps any others.

In the present case, M. Blandin was of opinion that the pleurisy was the result or consequence of a metastatic abscess at the base of the lung.

Pathologists have differed in their ideas as to the early changes, which an inflamed vein undergoes. M. Cruveilhier is of opinion that, as soon as a vein is fairly inflamed, the circulation through it ceases, that a clot is then formed within, and that, when suppuration ensues, this clot or coagulum is more or less completely dissolved in the purulent matter.

MM. Gendrin and Tessier agree so far with M. Cruveilhier in believing that the circulation of the blood is arrested in an inflamed vein, and that a clot is formed in consequence within its cavity; but they suppose that the pus is not secreted from the lining membrane of the inflamed vessel, but that the coagulum itself suppurates. All this however seems to be mere conjecture; and M. Blandin alludes to a case, which occurred in his practice at the Hospital Beaujon some years ago. It was that of a man, who was bled in the median basilic vein for a fracture of the ribs. Phlebitis supervened; and the patient died before pus had time to form.

On dissection, neither pus nor blood was found within the vein; but the vessel was observed to be strongly contracted upon itself. When however the system exhibits the symptoms of purulent infection—such as frequent shiverings and typhoid depression—we believe that pus has, perhaps, invariably been absorbed into the mass of circulating fluid, especially if the phlebitis has been of traumatic, and not of spontaneous, origin. In the latter case, the progress of the morbid changes is more gradual and slow than when the accident has followed upon a wound of a vein; the circulation is only gradually arrested, in consequence of the obstacle to its free course from the layers of lymph, deposited upon the inner surface of the inflamed vessel, becoming greater and greater, in proportion as the quantity of this lymph increases. Such is the pathology of the *phlegmasia alba dolens*. The absorption therefore of pus into the mass of blood is not so direct and easy in spontaneous phlebitis, as in that form, which supervenes upon an injury of a vein; and hence the prognosis in the former case is perhaps not altogether so serious as in the latter.

M. Blandin explains the occurrence of the local or circumscribed visceral inflammations, which so frequently occur during the course of phlebitis, in the following manner. The blood, modified in its character by the admixture of pus with it, is much thinner and less readily coagulable than in a state of health. Hence there is a tendency to its exuding from the vessels. If this exudation occurs from the cutaneous veins, ecchymoses are formed, such as we observe in the progress of typhus and other malignant fevers; but if the viscera are the seat of the exudation, the effused blood acts like a foreign body, and gives rise to a subacute inflammatory action, which is local and circumscribed from its existing cause being limited to a spot. These exudations generally take place on the surface of the viscera; and hence it is that the secondary abscesses, which we meet with in the liver, lungs, &c. in cases of fatal phlebitis, are usually superficial and not deep-seated in the parenchyma of the organs affected.

These circumscribed deposits commence by a small brown-coloured spot or stain, which gradually extends and excites an inflammatory action around; and this occurs the more readily that there is evidently something *septic* in the effused or extravasated fluid, which hurries on, it would seem, the inflammatory to the suppurative stage.

From the preceding remarks it will be seen that we (M. Blandin) do not agree with those pathologists, who suppose that the internal or secondary abscesses in cases of phlebitis are the result of purulent matter being actually transported to, and deposited in, the parts affected. We have only to examine the parts, at different stages of the lesion, to satisfy ourselves that this doctrine must be incorrect. At first we perceive only a darkish-coloured ecchymosed spot; this spot gradually becomes of a deeper colour and more extended; the affected tissue is found to be much more friable than before; the centre of the spot passes gradually to a lighter-coloured greyish hue, and becomes softer and softer, until at length the disorganization, arising from the suppurative process, is fairly established.

The lungs are the organs most frequently affected with these secondary abscesses, and the right lung more frequently than the left one; then the liver, the spleen, the brain, subcutaneous and sub-aponeurotic cellular tissue, and lastly the kidneys and the muscles.

The synovial and serous membranes are occasionally, although more rarely, involved in the inflammatory action.

The treatment of phlebitis, and especially of the traumatic form, requires the most energetic depletory measures in its early stage.—*La Lançette Française.*

---

### M. CHOMEL ON TARTAR EMETIC IN PNEUMONIA.

This eminently practical physician of the Hôtel Dieu has not, it seems, so high an opinion of the tartar emetic practice in controlling thoracic inflammation, as many of his professional brethren in Paris. He frequently uses it; but only as a subsidiary remedy, after a decided impression has been made on the disease by blood-letting. With respect to its having any directly antiphlogistic or contra-stimulant properties, independently of the depression induced by nausea and by evacuation, M. Chomel professes himself to be very sceptical; and hence, of late years, he has discontinued the common usage of combining opium with it, for the purpose of inducing a *tolerance* of the antimonial. According to his views, its action is to be referred to an energetic revulsion upon the alimentary tube, and to the powerful compression of the lungs during the efforts of vomiting, aided by the nausea which precedes and follows these efforts.

The antimonial will always be found of most efficacy, when the first violence of the inflammatory attack is arrested, and when the disease indicates a tendency to remission or abatement.

Alluding to the excellent effects of intestinal derivation upon all thoracic inflammations, M. Chomel takes the opportunity of strongly recommending castor oil as the safest and one of the most effectual purgatives, which can be used for this purpose.—*La Lançette Française.*

---

### On Paralysis in the Insane.

(From a Memoir to which the Prize was awarded by the Societé d'Emulation.)

M. Bayle, in 1822, was the first who particularly described this very frequent complication of insanity. He attributed it to a chronic inflammation of the membranes of the brain.

It must be confessed, however, that this opinion as to its cause is perhaps as much to be traced to a rational conjecture, as to the results of actual observation; for, in not a few cases, no traces of meningitis can be any where discovered. M. Calmeil, although a decided anatomo-pathologist, has therefore, in his work *De la Paralysie considerée chez les Alienés,* carefully avoided giving any express opinion on this subject. In the present state of our knowledge, it will be wise to follow his example.

Paralysis, in some form or another, is of very frequent occurrence amongst the insane. Whoever has frequently visited lunatic establishments cannot fail to have observed the number of the unfortunate inmates, who are afflicted with some lesion of motion in one part or another of the body.

Many, for example, are noticed to stammer in speaking; others incline over more to one side than to another in walking; a third set are paralyzed in their upper or in their lower extremities; and lastly, a few are found to have lost all feeling and mobility on one entire side of their bodies.

These various forms and degrees of palsy are very generally found to be connected with some organic lesion of the nervous centres, such as ramollissement or induration of their structure, abscesses, effusions of serosity, tubercles, and lastly, we may mention tumors and other diseases of the inner table of the cranium itself.

The symptoms of general palsy in the insane may be described as follow.

The earliest symptom is generally a stammering or some confusion of speech. The invalid cannot pronounce certain words, or he hesitates and falters before pronouncing them. As yet, perhaps, there is no visible disturbance of the muscles of the face; and if the patient be ordered to put his tongue out, no irregularity or deviation in its movements can be detected. The practised physician however will often be able to detect some faint signs of incipient paralysis, which altogether escape the attention of the less experienced.

The prognosis of insanity is always much less favorable when any form of paralysis has made its appearance, than it may have been previously.

As the confusion in the speech increases, the irregularity or immobility of certain muscles of the face becomes more obvious and decided; the looks acquire a dull and heavy expression; the arms lose much of their former strength and energy, and at length the lower limbs also indicate an increasing feebleness and want of steady support. We have said that the upper extremities are usually affected before the lower. Now this statement may seem to be contradicted by the experience of many excellent authors, who in their writings allude to the instability and tottering gait of patients, during the earlier stages of general paralysis, while the arms retain their normal power.

Medical men are, however, apt to be somewhat deceived upon this point, from forgetting that the lower limbs have the weight of all the body to support, and will therefore announce at once any weakness or want of energy, while, all the time, the upper extremities, although quite as much enfeebled, will not manifest any loss of power. M. Calmeil very justly reminds us that, in order to judge accurately of the relative condition of the upper and of the lower limbs in cases of incipient paralysis, we ought to examine the patient when in the horizontal position, and notice the freedom and vigour of movement of the legs, when relieved from the superincumbent weight of the body.

M. Lallemand, in his admirable letters on the diseases of the encephalon, has alluded to the same view of the subject in these words: 'In conclusion, the incomplete general paralysis of the insane *appears to commence* in the pelvic extremities, because the slightest weakening of these members is more readily observed, in consequence of the force required for the exercise of their functions.'

M. Rodrigues, the author of the present observations, is inclined to believe, from the result of his own observations in the lunatic hospital at Montpelier, that, in the general paralysis of the insane, the arms are usually more affected with weakness than the lower extremities. He alludes more particularly to the case of a female patient, who could not carry her hand to her mouth, although she continued to walk perfectly well.

*Second Stage.*—The speech has become more embarrassed and confused, the patient being often unable to do more than answer in monosyllables. When he attempts to rise, he is obliged to rest upon his hands, and the exertion is tedious and difficult. The gait is unsteady, one limb being dragged after the other, and the slightest slip causing the patient to fall. In the horizontal position, however, the lower limbs may still exhibit much more vigour and freedom of movement.

The whole muscular system is affected, more or less, with the paralytic weakness; the muscles of the neck, trunk, and abdomen, and even some of the internal muscles, as those of the pharynx, bladder, rectum, &c., participating in the general atony.

The sensibility, too, of the system, general as well as special, is usually impaired at the same time. This indeed is the case in almost all palsies, which have their seat in the encephalon: the intelligence is first affected, then the motility, and lastly the sensibility. When the palsy is owing to any lesion of the spinal marrow, the integrity of the mental functions is not affected. In the second stage of paralysis, occurring in the insane, the sphincters of the rectum and urinary bladder generally lose their normal contractility; and hence the miserable patient is too often the victim of sad filthiness.

Although the prevailing state of the mind is usually that of taciturn imbecility, there occur occasionally fits of excitement, during which the speech, previously most difficult and incoherent, may recover a perfect freedom and readiness of utterance. It would seem as if the state of general irritation had awakened, for a time, the slumbering action of the cerebrum from its state of apathy and stupor.

Although the powers of mastication and deglutition are generally more or less impaired, the appetite is not unfrequently not only good, but even voracious. Cases have occurred, where the patient, in his haste to swallow a large morsel, has had the misfortune to let it drop into the rima glottidis, and been quickly asphyxiated. M. Bayle narrates two cases of sudden death, where on dissection the larynx was found plugged up with food.

The digestive and assimilative functions often retain their normal activity, and the state of the circulation and breathing too may be perfectly healthy all this while.

*Third Stage.*—The paralytic weakness is now considerably advanced. The intelligence is *nulle*. The patient scarcely ever speaks, and if he does, it is only confusedly in monosyllables; his limbs totter or give way under him, and he is unable to raise himself from bed. The senses of vision, hearing and smelling are all much impaired; and, although the appetite often remains vigorous, there seems to be little or no discrimination of taste.

During this stage, some bowel-affection, and most frequently a diarrhœa, usually comes on, and rapidly exhausts the patient's strength. Eschars form on those parts on which he lies, and death soon closes the distressing scene of misery.

The age, at which general paralysis in the insane is most frequently observed, is from the 30th to the 50th year of life. It would seem that the sanguineous temperament predisposes to its occurrence. Hypertrophy of the heart—generally of its left ventricle—has been detected in many of the unfortunate victims of this form of palsy.

The morbid state, on which the disease is in most cases connected, is a chronic inflammation of the cerebral meninges, which has terminated in serous effusion.

At other times, it is found to be a general hardening of the cerebral mass, or purulent deposits in, or tuberculous vitiations of, its tissue; or a ramollissement of one or of both hemispheres, &c.—*Revue Medicale.*

---

### On the Diseases of Old Age.

The data, from which M. Prus has derived his conclusions, were obtained from the histories of 430 patients, who died at the Bicetre during the period of four years. It will be necessary, however, to deduct forty of these cases from our list, as the patients had not attained 60 years of age. The remaining 390 are arranged by M. Prus in the following manner:

| | |
|---|---|
| 149 | died from diseases of the respiratory organs. |
| 101 | ——— diseases of the nervous centres and their coverings. |
| 64 | ——— diseases of the organs of circulation. |
| 49 | ——— diseases of the digestive tube. |
| 8 | ——— diseases of the liver and its appendages. |
| 19 | ——— diseases of various kinds. |
| 390 | |

It has frequently been supposed that the most common diseases of old age are those of the abdomen. This idea is quite refuted by the preceding table; the cause of death is much more often in the chest and encephalon.

M. Prus then gives another table of those patients who were successfully treated during the same period of time, when the preceding fatal cases occurred.

Of 685 cases, which were either cured or greatly relieved at the Bicetre,

| | |
|---|---|
| 216 | were diseases of the respiratory organs. |
| 151 | ——— of the nervous centres. |
| 144 | ——— of the digestive tube. |
| 54 | ——— of the organs of circulation. |
| 22 | ——— of the skin. |
| 98 | ——— of various kinds. |

According to this table, the stomachic and bowel affections were considerably more frequent than those of the circulatory system.

M. Prus, in alluding to the peculiarities of disease in old age, very forcibly dwells upon the frequent feebleness or defect of reaction in the diseased organs, and on the obscurity and uncertainty of diagnosis which are consequently induced.

Thus the lungs may pass into the state of grey induration, or the stomach may be the seat of cancerous degeneration, without the necessary existence of any well-marked symptoms during life. Even the heart itself, as Bichat remarked, may be most seriously altered by disease in old age, and yet perhaps not even a suspicion of this being the case may have been once excited during the life of the patient. This circumstance shows the high importance of a strict examination of the thoracic organs in aged patients, whether there be or be not any symptoms of cardiac disease.—*Archives Generales.*

---

### M. Rostan's Remarks on Clinical Medicine.

One of the favourite axioms of this distinguished Professor is that *there is no*

*lesion of function without a coexistent organic lesion.* Cases do indeed occur, now and then, where the pathologist fails in detecting any mell-marked deviation from healthy structure, to account for the death of a patient; but the *apparent* absence of any change is very probably owing only to partial or defective examination.

M. Rostan, in continuation, remarks: *we cannot expect to discover the organic lesion after death in many of the Neuroses*; *for the lesion is transitory and evanescent; and this is necessarily so, because the functional derangements are themselves transitory and evanescent.*

Our sensations themselves impress real and material modifications upon our organs of sense: thus sound causes an organic modification in that part of the brain, which presides over the sense of hearing, &c.

(This doctrine, it will be observed, is strictly phrenological. We might suppose that our author is a stanch disciple of Gall.)

Further on, M. Rostan observes:—

"The brain is the seat of the soul, which probably resides in the cortical or cineritious part. It is a multiple organ; but we cannot localise the different faculties of intelligence any more than we can say what is the part of this organ which presides over the movements of such and such members."

(Here we find that the Professor rejects the very basis of phrenology.)

But leaving such metaphysical questions, let us hear M. Rostan's opinions on matters of more immediate practical import.

In reference to the general treatment of diseases, he boldly announces that '*all medicine consists in diagnosis.*' He illustrates this position by discussing the common disease of Amenorrhœa.

The various derangements of menstruation are not specific diseases, but are rather functional maladies, indicative of the lesion of some distant organ or another. In one patient, amenorrhœa is attributable to an organic affection of the uterus; in another to some disease, as tubercles, of a more distant organ; in a third to a defect of sanguification; in a fourth to an excess in this function; and in a fifth to some cerebral irregularity.

If such be the case, how can we expect to treat the disease successfully by having recourse to one uniform mode of prescription, without regard to the primary cause, on which the morbid action depends?

Dropsy is another disease which is rarely if ever essential or idiopathic. The effusion of water is only a symptom of an organic lesion existing somewhere; and hence our remedies ought to be directed against the *cause,* and not against the *effect.*

*The medicine of symptoms is,* says M. Rostan, *the worst of all medicines.*

This doctrine, however just as a general principle, may be carried to a pernicious extent in so uncertain an art as that of physic. The wisest and most experienced practitioners find it not unfrequently necessary to be satisfied with trying to relieve symptoms, in cases where the primary and essential cause of the disease is obscure and uncertain.—*Journal des Connoiss. Med. Chir.*

---

ON THE APPLICATION OF ARITHMETIC TO THE RESULTS OF THERAPEUTICS. By M. DOUBLE.

M. Double is one of the oldest and most experienced members of the profession in the French metropolis, and being a man of high repute and having never attached himself to any particular sect or school in medicine, his remarks on any professional subject cannot fail to deserve our studious attention.

Our readers are probably aware that, of late years, there has been an energetic controversy among the medical men in Paris as to the value of numerical calculations, (or, as it is sometimes very absurdly called *statistique,*) in ascertaining the efficacy of any given mode of treatment in disease. For example, there was, within the last twelve months, a very keen discussion in the Royal Academy of Medicine as to the relative value of the purgative and of other methods of treating typhoid fever. To settle this practical question, an appeal was attempted to be made to lists of cases treated in different manners; one physician insisting upon the predominance of his list, and another upon that of his. In short, the Academy could not come to any decided opinion on the subject, and, like a prudent and cautious arbiter, announced their judgment that the question was still open to further investigation!

M. Bouillaud, on this as on most other topics of medical enquiry, carries his opinions to an extravagant length. In his recent work on Medical Philosophy he descants, in most flowing language, upon the wonderful changes to be effected in our science by an adoption of the *calcul statistique*, and, as a matter of course, he adduces the astonishing results of his *saignées coup sur coup* in the treatment of fever, pneumonia, &c. to confirm the justness of his doctrines. But M. Bouillaud, although a clever and enterprising man, is much too hasty in his adoption of certain views, and much too energetic in his advocacy of them, to gain general credit among experienced men. We shall not however anticipate the remarks of M. Double, but proceed to give an analysis of a very valuable paper, which he recently read before the Royal Academy of Medicine.

After some preliminary remarks on the value of public discussion, the worthy old gentleman comments, in a playful and quietly quizzical style, on the prevailing fashionable partiality to the science of numbers.

" Statistics is now-a-days, among the various branches of human knowledge, a science quite *à la mode*. In the ardour of its zeal, in most respects very praiseworthy, it mixes itself up with everything, and would willingly seize upon all. It very quickly made an irruption upon *notre pauvre medecine*, which on most occasions is but too ready to give admission to every eccentric notion, which is presented to it. Not satisfied with the usual methods, which are slow, difficult, and without any eclat or glory, the ardent spirits amongst us have, on all occasions, sought to find out new ones, which promise a quick solution of every problem. It is thus that, at the present moment, the *Statisticiens* of medicine are trying to substitute mathematical for logical analysis, and to supplant mere reasoning by calculation and arithmetical induction."

Now the object of this arithmetical induction is to ascertain, from the examination of a multitude of cases, the method of treatment best fitted to subdue a particular disease; just in the same manner as the mathematician calculates, from the observation of numerous facts, the probability of future events. But it would seem to be forgotten by those, who draw this analogy, that the *facts* of the latter are uniform and exact, whereas those of medical science are only so many detached data, isolated and not general; in other words, no two cases of the same disease are perfectly alike in all their characters. And indeed, as M. Double well observes, unless this were the case, the art of medicine would be reduced to the same level as any of the common trades of the artisan.

If, for example, it could be unhesitatingly pronounced that any given disease is to be treated in a certain manner—let us say by three or four bloodlettings, the application of so many leeches, and so forth—without reference to its existing type, the constitution of the patient, &c., it is obvious that almost any one might attend such a case as profitably as the most experienced physician.

Well may M. Double playfully remark that all such attempts to reduce the practice of the medical art may be compared to the proposal of a shoemaker to take the medium size of foot in a thousand persons, and then to *chausser tout le monde* upon this one measure.

As long as each case of a disease is an individual problem, which must be examined by itself, and not merely in reference to any antecedent cases of the same disease in different persons—and we believe that all really practical physicians will coincide in opinion on this subject—we cannot expect that the doctrine of mathematical calculation can ever be strictly applicable to medicine.

The numerical method, applied as a rule to guide the physician in his practice, is extremely apt to sanction one of the most serious errors in therapeutics —to wit, the adoption of absolute and exclusive systems of treatment.

This error necessarily springs from supposing that a disease is a uniform, fixed, and invariable phenomenon—a doctrine utterly irreconcileable with what we observe in nature.

Far from this being the case, we should say that a disease is a variable series of unsteady acts, which are changing every day, nay, every hour of its existence. The *pneumonia* of to-day is not the *pneumonia* of yesterday; neither is the *pneumonia* of Paul the same as the *pneumonia* of Peter.

Consider the embarrassment and disappointment which the young physician almost always experiences when he passes from the lecture-room of the professor to the sick-room of the patient. In the former he was completely *au fait* with the whole book of nosology; in the latter, alas! he finds his knowledge very speedily at fault. Now all this arises from the marked contrast that exists between the uniformity and exactitude of oral or written descriptions of disease, and the variable characters which it exhibits in actual practice.

If we examine those classical collections of cases, which are so often appealed to as faithful transcripts of accurate observation—we allude to the works of Hippocrates, of Baillou, the letters of Morgagni, the consultations of Hoffman, the ratio medendi of Stork, Dehaen and Stork—do we not find that the descriptions of the same disease are by no means uniform in the pages of these great authors?

Now if a disease is not uniform in its character or symptoms, the same method of treatment cannot possibly be proper for all cases without exception.

Let us take for example the diseases known by the name of Typhoid fever; and this is probably the most appropriate example that we can select, as it is upon the treatment of this very malady, that the question of the 'numerical method applied to therapeutics,' was first mooted.

M. Double takes the present opportunity of utterly disclaiming all assent to the various pathological names which have, within the last five and twenty years, been imposed upon typhoid fever: "depuis long-temps je gemis sur cette nouvelle denominations de fievres typhoides." Sydenham, he goes on to state, has most truly said that improper appellations of diseases have introduced into practical medicine more errors than even false systems and theories.

Since the period of M. Broussais's first publication, a most pernicious custom of grouping together all continued fevers, from the simple *embarras gastrique* to the most aggravated *fievre ataxo-adynamique*, under one general denomination; as if they were all mere varieties or degrees of one morbid state.

Now we are quite convinced that such generalisations are based on a most grievous error;—the error of regarding that pathological condition, known under the term of *typhoid or malignant fever*, as a distinct, independent, and essential form of disease in itself, instead of viewing it as a mere morbid state of the system, which is frequently co-existent with, and forms a complication to, other maladies.

Who, for example, has not met with cases of ataxo-adynamic pneumonia, or in other words, of pneumonia complicated with a typhoid state of the system?

The peritonitis and uterine phlebitis of puerperal women, small-pox and the other exanthematous fevers, extensive burns, surgical operations, &c., often terminate fatally from the accession of typhoid symptoms. In the same manner gastric disturbance, and the various varieties of bilious, catarrhal, and inflammatory fevers may have a typhoid character grafted upon, or superadded to, them, so that their proper features or symptoms are greatly obscured. I have never seen, says M. Double, typhoid fever without the coexistence of some other morbid state; and hence I cannot admit it as a distinct and independent disease. In all cases, without exception, some nervous disturbance with or without febrile reaction, or gastric irregularity, or an inflammatory or bilious affection, precedes and initiates, so to speak, the development of the typhoid phenomena.

It is one of the evils of the present medical epoch, that physicians study disease almost exclusively in hospitals, and neglect far too much the study of its numberless forms in private practice. In hospitals, let it be remembered, that diseases are rarely observed except only when they are fully developed. Hence the phenomena of the earlier stages—the stages of incubation and of invasion—are almost totally overlooked, and attention is paid only to the existing symptoms of a disease already fairly established. Again too, the stage of convalescence can never be accurately watched, throughout its whole progress, in hospital practice; and hence we can never have a sufficient proof of the entire recovery of our patients.

It is from the partial and limited view of diseases, as observed in public institutions, that not a few of the prevailing errors in medicine have sprung up.

As far as regards myself, the more that I (M. Double) see of disease, the more I am convinced that each case forms a new and almost isolated problem for my study. For the nicety and tact of practice do not consist in the mere detection and recognition of certain symptoms, but in the accurate and comprehensive review of all the peculiarities of each individual patient—his corporeal and moral constitution, his idiosyncrasy, his age, the season of the year, the prevailing character of the weather, the climate and influence of locality, profession, pursuits, cares, &c.

If therefore each case of disease has something special in itself, and is marked by a difference, more or less decided, from all other cases, how can we think of applying the vigorous inflexible method of arithmetical calculation to the results of medical practice, to discover any uniform method of treatment?

Every exclusive theory in medicine is a *non-sens* of pathology; and every absolute method of treatment is a *contre-sens* of therapeutics.

Montesquieu, after his profound meditations on the public organisation of societies, came to the conclusion that political as well as moral good is always found to exist in a *juste milieu;* and in like manner we may say that a therapeutic truth 'se trouve toujours dans de moyens termes.'

It is from a conviction of the value of these principles that, during my long professional life, I have always followed a rational and enlightened *eclectism,* based upon a comprehensive analysis and induction—a system or creed, which may be defined to be *the logic of facts enlightened by the logic of reflection.*

In conclusion, Mr. Double enunciates the three following propositions.

1. Individuality constitutes an immoveable truth in pathology : a disease is not a simple, fixed and uniform entity, but is rather a series of diverse, irregular, and changeable acts. Hence all exclusive systems in medicine must be based in error.

2. The strict application of numerical calculation or statistics to therapeutics is impossible.

3. The only admissible methods are logical analysis and induction.—*Gaz. Medicale de Paris.*

---

### Obstinate Ague, complicated with Ascites, &c. cured with large Doses of the Carbonate of Iron.

A youth, 14 years of age, had been in ailing health for several years, when Dr. Gimon was consulted. It appeared from his parent's statement that, when he was in his fifth year, he was attacked with a quotidian fever, which returned regularly every day for three months successively. It resisted every remedial means employed, and only ceased after an attack of severe diarrhœa.

In the course of the following Spring there was a return of the intermittent fever; and at this time it assumed a quartan type.

It would seem that the patient never fairly got rid of this most troublesome complaint for several successive years; and, as might be expected, his general health had in consequence suffered greatly from this protracted illness. When Dr. Gimon first saw him, he found him pale, weak, and dropsical; the spleen was enormously enlarged, projecting far beyond the edges of the ribs, and occupying nearly three fourths of the abdominal cavity, which was partly filled with water; the limbs were œdematous; the urine was scanty; there was a most distressing dyspnœa, aggravated upon any motion or exertion, and the appetite and powers of digestion had long been weak and uncertain.

M. Gimon, attributing the dropsical and asthmatic symptoms to the enlarged state of the spleen, had recourse at once to the use of large doses of the sulphate of quinine, with the view of reducing this disease.

This treatment was persevered in for six weeks, without however any decidedly beneficial effects being obtained. Not only was the size of the spleen little, if at all, diminished, but even the return of the agueish paroxysms was not very sensibly arrested.

The doses which Dr. G. administered were immense—nearly a drachm a day! He alludes to the opinion of M. Bailly, that the stomach, in patients affected with enlargement of the spleen, will usually bear larger doses of medicines than in almost any other case.

The quinine being omitted, no further medication was resorted to for six or eight weeks; but then, the state of the invalid becoming more serious, Dr. Gimon was again consulted. He now recommended the use of the carbonate of iron, along with a weak decoction of cinchona. The dose of the carbonate was rapidly increased from twelve grains to two drachms in the course of the twenty-four hours.

This treatment was persevered in for several months; and ultimately with the happy effect of completely removing all signs of disease: the size of the spleen had become remarkably reduced; the dropsical effusion had entirely disappeared, and the disposition to the returns of intermittent feverishness seemed to be quite overcome.

The cure was moreover permanent.

*Case* 2.—A countryman, 21 years of age, of a lymphatic constitution, had been affected with a quartan ague for upwards of three years, when he first consulted Dr. Gimon. His complexion was pale and yellowish; the features were puffed and bloated; the limbs were œdematous; the abdomen was swollen, especially over the region of the spleen; and his general health was frail and uncertain. For the preceding six months, the ague had returned regularly every third day: and during the intervening days, or those of apyrexia, the patient's strength was so feeble that he could not engage in any occupation. Although the tongue was red* and the epigastrium was rather tender on pressure, Dr. G. did not hesitate to have recourse to the use of the quinine—10 grains every six hours. This was continued for eight days; but the paroxysm of the disease were not arrested.

The carbonate of iron was now substituted—at first in small doses, which were rapidly increased, so that at length the patient took six drachms in 24 hours. For the first fortnight of this treatment, no very sensible amendment took place, the paroxysms of the fever returning as frequently and as severely as heretofore. After this time, however, the state of the patient began to improve, and the fever and other symptoms gradually subsided. In the course of three months, the enlargement of the abdomen had quite disappeared, and the health was completely re-established.

These two cases are fair examples of the good effects of steel in some obstinate and long-continued agues. It is not a new practice, as we find that it

* M. Bailly has very properly remarked that the mere redness of the tongue, even although accompanied with a certain uneasiness or tenderness of the epigastrium in many agueish cases, is not a sufficient contra-indication to the use of bark. Unless there is a febrile irritation of the system at the same time, we should not attribute these symptoms at once to a gastritic affection.

has been recommended at different times by several authors. The writer of the article *fievre*, in the Dictionnaire des Sciences Medicales, mentions a case of a quartan fever, of four years' standing, cured with the sulphate of iron, after it had resisted a host of previous medicines.

We have already said that the mere circumstance of redness of the tongue and uneasiness of the epigastrium need not preclude the administration of steel. —*Journal des Connoissances.*

---

### Treatment of Epilepsy with the Nitrate of Silver.

The late Professor Carron treated a great number of cases of epilepsy with very marked success, by means of the nitrate of silver internally administered. Were practitioners sufficiently aware of the admirable effects of this metallic salt, we are satisfied that they would have recourse to its use more uniformly, and not lose time in trying various other less potent remedies.

When Joseph Frank published an account of his visit to Paris and London, he had numerous conferences with M. Heim, who first proposed the employment of the nitrate in the treatment of epilepsy. The formula, which he recommended, was—

℞. Argenti nitrat. gr. x.

Opii puri . gr. vj.

Extract. conii, ℨij.

Extract. glycirrh. ℨj. Misce.

Divide into pills of three grains each; three to be taken on an empty stomach every morning.

The English and American physicians appear to have obtained great success in the treatment of epilepsy from the use of these pills.

In the medical journal, edited by MM. Corvisart, Boyer, and Leroux, for 1814, we read the following paragraph in a communication from Dr. Valentin: "I am informed that in the United States of America they continue to employ the nitrate of silver in cases of epilepsy: numerous examples of its successful administration are recorded in their journals."

The late Professor Carron cured more than 20 cases of genuine epilepsy by means of the nitrate: in some the treatment was very tedious, as it required to be persevered in for a full twelvemonth.

The usual dose for an adult, at first, is the eighth or so of a grain, twice a day. This dose should be gradually, but slowly, increased. With respect to the occasional discoloration of the surface from the internal use of the nitrate of silver, he (M. C.) never met with a single case of it.

The following two cases are illustrative of the efficacy of the remedy.

M. Laurent, now a physician resident in the environs of Lyons, was attacked with epileptic fits in the twelfth year of his age. He was treated with valerian, bismuth, copper, &c. &c.; but the disease obstinately resisted every remedy employed.

Professor Carron, being now consulted, recommended the use of the nitrate of silver—the 20th part of a grain evening and morning—and the introduction of a suppository, containing valerian, assafœtida, and opium, every night at bed-time. The youth was, at the same time, advised to discontinue all his studies, to live very abstemiously, and to take a bath three times a week. The fits returned every fortnight. After three months' treatment, their recurrence was once a month; after six months, once every seventh week or thereabouts; and, after a twelvemonths' use of the remedy, there was a complete cessation of the fits. The use of the salt was now intermitted for two months; but renewed for some time, in order to secure greater certainty as to the result. During the following eight years, the patient took the nitrate for two months each year. There has never been any return of the disease.

*Case* 2. Ernest B., 10 years of age, had been for several years subject to epileptic fits, in consequence of which the left eye exhibited a partial squinting. The fits usually recurred once a week or so; and their approach was generally announced, for two or three days previously, by the greater obliquity of the left eye, and dimness or loss of vision in it.

Professor Carron recommended the employment of the nitrate of silver. Its use was continued for three months, without any sensible improvement in the condition of the patient; but after this period, there was a very marked retardation in the return of the fits. From the fourth to the sixth month there occurred only one fit; and from the sixth to the ninth only two, both of which took place within ten days of each other. After eleven months' use of the remedy the fits appeared quite subdued, and the strabismus of the left eye had almost entirely vanished.—*Bulletin General de Therapeutique.*

---

### Observations on the Poisonous Effects of Rue, and on its Influence on the Uterus.

It would seem that the various means deemed capable of inducing abortion are not equally made use of in all countries alike; each country appearing to follow some particular practice in preference to any other. In Paris the puncture of the membranes is generally resorted to; and it is truly disgraceful to think that not only many midwives, but even some medical men, lend themselves to this flagitious practice. I lately saw a melancholy case of a young woman, who died of *metro-peritonitis* in the Hôtel Dieu of Paris, from a deep penetrating wound of the uterus, induced by the use of a trocar for the purpose of bringing on miscarriage.

In other parts of France the use of rue and of savine leaves, in various preparations, is chiefly trusted to as provocatives of abortion. At present we shall confine our remarks to the former of these two plants.

There has been much difference of opinion as to the medicinal effects of rue; some alleging that it exerts a direct and immediate action on the uterine system, while others contend that, whenever it seems to do so, this action is only secondary to, and consequent upon, an irritation of the intestinal canal and a disturbance of the nervous system; and hence that its use much oftener fails in provoking miscarriage than succeeds.

To determine the question, Dr. Helie, the author of the present observations, reports several cases. One of these is as follows.

A young female, having suffered a great deal in her first accouchement, was resolved to try some means to bring on abortion in her second pregnancy. She applied to Dr. Helie for the purpose, being about four months advanced; but he very properly declined giving her any advice, but that of dissuading her from her intentions. She told him, however, that, if he would not prescribe for her, she would apply to some other person. A fortnight afterwards she returned to him; and then she was no longer pregnant. She mentioned to him that, by the advice of a woman, she had taken three fresh roots of rue, cut them in pieces, and then boiled them in a pound and a half of water down to three small cupfuls, which she drank one evening on going to bed. Dreadful pain in the stomach came on, accompanied with vomitings, and with such universal oppression, that she thought she was dying.

This state continued all the night, and next day the symptoms were much abated. But now she began to experience colicky pains, slight at first, but gradually increasing in severity, and returning at intervals. On the evening of the second day, they became much more violent, and were evidently the pains of labour: abortion came on soon afterwards—in 48 hours after taking the decoction of rue.

*Case* 2. A young woman, residing in a farm-house, was suddenly seized with most severe vomiting, violent twisting pains in the abdomen and limbs, restlessness, and tendency to delirium. She had all the appearance of being intoxicated.

Dr. Helie suspected that the illness was the effect of medicines, which had been taken with the view of provoking abortion: the patient seemed to be in the seventh month of pregnancy, although she positively denied it.

He therefore contented himself by withdrawing certain drinks which she had been using, and by confining her to simple barley-water.

The vomitings speedily ceased; but the abdominal pains continued to increase, and in the course of the following day, she was delivered of two infants.

Alarming symptoms of poisoning came on afterwards; but these fortunately subsided by degrees, and the girl ultimately regained her health. She afterwards admitted that she had made use of a strong decoction of rue leaves.

*Case* 3.—A girl, in the fourth month of pregnancy, took for several days a strong dose of the fresh juice expressed from rue leaves. Vomiting, severe colic, great prostration, and tendency to syncope, somnolence, delirium and coldness of the whole surface came on. There was also, as in the preceding case, an inflammatory swelling of the tongue, accompanied with a profuse salivation. The expulsion of the fœtus did not happen till the sixth day after the swallowing of the poison. The acrid-narcotic effects did not cease altogether for another week.

From the particulars of these cases, we may reasonably conclude that rue, in powerful doses, has decided narcotic irritant effects. Its action in diminishing the force and frequency of the heart's movements appears to be as marked as that of digitalis: in some cases, under its influence, the pulse has fallen to thirty beats in the minute.

Its peculiar action on the tongue, inducing an active inflammation of this organ, deserves notice.

Dr. Helie is of opinion that it also has a direct and *elective* action on the uterus, and that therefore it may be regarded as a provocative of miscarriage, quite independently of its irritant and narcotic effects on other parts of the body.

If such be the case, we may reasonably expect that the use of rue will be serviceable in many cases of amenorrhœa. The fresh plant is very much more powerful than the dried one: indeed we should never trust to any preparation of the latter. The expressed juice of the fresh leaves is the most active form in which in can be taken; and, after it, a decoction of the fresh plant.—*Annales d'Hygiène, &c.*

---

### Researches on Menstruation.

M. Petrequin, one of the most intelligent contributors to the Bulletin Medical Belge, published lately a small work in which he has recorded the result of his enquiries on the above subject.

The first question he proposes for consideration is, at what age does the menstrual flux usually appear in our climate (France)? Two hundred and seventy-two cases have served him to draw up the following table, from which it appears that the earliest age is about ten years, and the latest about twenty-two years:—

| | |
|---|---|
| 4 at | 10 years of age. |
| 10... | 11 |
| 15... | 12 |
| 33... | 13 |
| 33... | 14 |
| 45... | 15 |
| 48... | 16 |
| 32... | 17 |
| 27... | 18 |
| 12... | 19 |
| 7... | 20 |
| 5... | 21 |
| 1... | 22 |

M. Petrequin, therefore, fixes upon the period between 13 and 15 years of age, as that at which puberty generally occurs in France.

From his researches it appears, that the more tardy that the first appearance of menstruation is, the more apt is the function to be irregular and disordered afterwards.

The next question, which our author endeavours to solve, is, at what period of life does the cessation of the catamenia usually take place? He fixes it at between the 35th and 55th years. It is well known that, in some females, it is prolonged considerably beyond the latter period. Thus Desormeaux has known it to continue to the 65th, Richerand to the 70th, and Gardien to the 75th year of life.* Occasionally the flow returns, after it has ceased for several years.

From the comparison of 60 cases, M. Petrequin states, that menstruation ceases between 35 and 40 years of age, in about one-eighth; between 40 and 45 years in one-quarter; between 45 and 50 in one-half; and between 50 and 55 in one-eighth of the whole.

We shall now briefly consider, whether the common notion that the epoch of life, at which the function of menstruation usually ceases, is really a very critical one to women, in reference to mortality.

M. Petrequin has not been able to satisfy himself on this topic from personal researches; he appeals, therefore, to the statements of preceding authors.

According to Muret, the period from the 40th to the 50th year of life is *not* more critical to women than the period from the 10th to the 20th year. M. Lupecq found that of 1,478 deaths in persons between 20 and 50 years of age there were 718 females and 760 males; and M. Benoiston states that, from the result of his enquiries, it appears that the period of life between 40 and 50 years of age is in truth more critical for men than for women.

M. Lachaise has come to the same conclusion in his Medical Topography of Paris.

Mr. Finlayson states as the result of his numerous researches that, after the period of infancy, the life of women is, on the average, considerably more lengthened than that of men.

It is not to be denied that at the period of life, when the catamenia cease, there is a tendency in some women to the development of certain diseased actions; but then be it remembered at the same time that in others—as in those who have long suffered from excessive or irregular menstruation—there is a very marked improvement of the general health: the one set of cases may therefore be said to be counterbalanced by the other set.

A few hygienic precautions, such as the use of cooling aperitive medicines, the use of light food, the abstinence from venereal pleasures (which are apt, according to Desormeaux, to induce cancerous disease), and the occasional loss of a little blood, will lead most women safely through this often-dreaded period of life.

We may notice, *en passant,* that blindness from amaurosis is of frequent occurrence, when the catamenia cease to return.

With respect to the quantity of the catamenial secretion, we may probably state it at about from three to five ounces. It is usually more copious in Spring than in other seasons of the year; and there is reason to believe that the sexual passions, and also the aptitude to conceive, are greatest in the former season. M. Villermé has deduced, from the comparison of 13,903 cases of labour, that most conceptions take place in the months of April, May, and June.

The quantity of the catamenial discharge is very generally greater in women of a voluptuous than in those of a cold and less susceptible constitution.

It is an idle waste of time to endeavour to find out the cause of the monthly return of the catamenia. All that we can say is that it is a law of the system in the human female; just as the period of nine months is that of utero-gestation, or as certain plants flower in certain months and not in others. The *why* of these phenomena is beyond our research; and he, who attempts to discover it, will only subject himself to the satire of another Moliere: *Opium facit dormire, quia est in illo virtus dormitiva.—Bulletin Medicale Belge.*

* The Belgian journals, a short time ago, mentioned the case of a woman giving birth to a child in her 70th year.

### CASE OF REMARKABLY PRECOCIOUS MENSTRUATION.

Dr. Susewind has related, in the April number of one of the German Journals, the case of a child now 27 months old, in whom there has been a regular return of a sanguineous discharge from the vagina every month since the end of the first year of her age. It usually lasted for two days, and exhibited all the appearances of ordinary catamenia. The genital organs are developed in a very extraordinary degree; the labia are full and prominent, and also covered with hair, the mammæ are as large as a good-sized apple, and the nipples are surrounded with distinct areolæ.

There are many similar cases recorded, or alluded to, in the works of Haller and Ploucquet; and since their time we find other analogous instances in most of the periodical publications. (We must refer the curious reader to the original paper for reference to these authorities.)

Lobstein and Canes each relates a case, in which menstruation commenced in, and continued regularly after, the second year of age: in one of these cases the girl conceived in her eighth year.—*Wochenschrift für die gesammte Heilkunde, and L'Experience.*

---

### Observations on Emmenagogues, and on the "Aura Menstrualis."

Dr. Krieg commences a very ingenious paper on the above subject, by endeavouring to shew that there is an *aura menstrualis*, or exhalation from the catamenial discharge of one female, which is capable of exerting a very decided influence on the uterine system of another female, so as to induce the menses in her, if they happen to be suppressed. He assures us that, in many cases of amenorrhœa, he has succeeded in obtaining a cure by simply recommending the patient to sleep with any other female, who may happen to be *unwell*.

He gives the particulars of some of these cases; but it is almost unnecessary to detail them.

[The hint appears to us (Rev.) to be worthy of notice, and deserves to be acted upon.]

Dr. Krieg, not satisfied with recommending the above simple expedient, suggests the trial in some cases of a direct inoculation with menstrual blood, by means of a sponge moistened with it and introduced into the vagina of the patient.

The remarkable resemblance between the smell of the *chenopodium olidum* and of the catamenial discharge has often suggested the employment of this plant in uterine affections; and from the multiplied testimony of numerous writers we cannot hesitate to recognize it as a potent emmenagogue.

About a century ago it was admitted under the name of *artiplex olida,* into the Pharmacopœias of London and of Edinburgh, in the form of a conserve or electuary. Dr. Kreig has recently tested the effects of the fresh juice, reduced to the state of soft extract, in several cases of amenorrhœa, and he assures us that it exerted very distinct emmenagogue powers.

In other cases, Dr. K. has found excellent effects from a combination of *secale cornutum, limatura ferri, and oleo-saccharum juniperi.—Caspar's Wochenschrift.*

---

### Proto-ioduret of Mercury in Syphilis.

A middle-aged man contracted a chancre in 1830: it was long of healing, and it seems that no regular course of medicine was taken at the time. For several years successively there appeared various symptoms of secondary syphilis. At one time he was treated with Dupuytren's pills, which consist of corrosive sublimate, opium and guaiacum; and at another with a solution of the sublimate and with fumigations of cinnabar. The symptoms gave way at the time; but re-appeared soon afterwards.

He was then treated with some preparations of silver, which within the last few years have been much recommended by Professor Serre of Montpelier; but these seemed to have no effect, although they were pushed to a very considerable extent.

The use of the proto-ioduret of mercury was substituted; and in a very short time the excellent effects of this remedy both on the general health and on the local disease were apparent. The cure was permanent; at least there was no return of the malady for a twelvemonth.—*La Lançette Française.*

*Remark.* The excellent effects of many of the preparations of iodine, either alone or combined with some mild mercurial, in secondary syphilis seem to be now admitted by all. We have found the common hydriodate of potash, and the Plummer's pill among the best of all the preparations.—(*Rev.*)

---

### On the Preparations of Silver in the Treatment of Syphilis.

Professor Serre of Montpelier, a corresponding Member of the Royal Academy of Medicine, published, about a year and a half ago, a pamphlet on the above subject.

Of late years, he says, a considerable modification of opinion as to the exclusively specific powers of mercury in the cure of the venereal disease has taken place, in consequence of the researches of different authors in France and in other countries. Some have substituted the employment of antiphlogistic remedies to the entire exclusion of mercurial medicines; others, more recently, have used certain preparations of gold with very decided benefit; and still more lately, M. Serre has given a trial to various salts of silver, at the large civil and military hospital at Montpelier. The preparations of this metal, which he has used, are the chloride, the iodide, the cyanuret, and the oxide.

Of the three first, the dose he commenced with was the tenth or eighth part of a grain; of the oxide he gave the fourth part.

He has published the reports of twenty-two cases of syphilis, in which the local symptoms were more or less severe, but in a very few only of which there was any decided constitutional contamination. In all, the treatment was quite successful. It is to be observed that no other medicines, of any sort, were administered at the same time.

M. Chrestien, in his researches on the action of the preparations of gold in the treatment of syphilis, suggested the employment of them in the way of friction upon the tongue. He assured us that in this manner they are more readily absorbed than in any other.

M. Serre has come to the same conclusions in reference to argentine salts.

The average total quantity of these salts administered by M. Serre may be stated at from six to twelve grains; but he is now of opinion that he was too timid in increasing the doses. The preparation, which he considers to be the most active, is the chloride; then the oxide, the iodide, the cyanuret, and, lastly, the pulverised pure metal.

With respect to the *modus operandi* of the salts of silver, M. Serre states that hitherto he has not been able to satisfy himself. He could not discover that there was any sensible increase or change in any of the secretions; nor was the breathing or circulation sensibly affected. The only appreciable effect produced was a slight and transitory excitement of the alimentary canal; but even this soon passed away, if the medicine was discontinued for a day or two. We must therefore confess entire ignorance on the subject; and this indeed is not very surprising, when we consider that, in spite of the innumerable attempts to discover the action of mercury in the cure of syphilis, we are nearly as ignorant as our forefathers were three centuries ago.

The advantages, which the preparations of silver have over those of mercury, are, according to M. Serre—1st, that they do not cause salivation, nor exert any hurtful influence on the digestive tube, and the lungs; 2d, that a great saving of expense, especially in hospitals, may be effected; and 3d, that the same precautions are not necessary, as when the system is under the influence of mercury.

M. Serre says that the salts of silver are much less irritating and exciting than those of gold, which are generally hurtful in nervous and susceptible constitutions, or whenever the chest is weak and delicate.—*Bulletin de Therapeutique.*

